# Why human societies adopt rigid moral rules: The efficiency–robustness trade-off

**DOI:** 10.1073/pnas.2535467123

**Published:** 2026-07-08

**Authors:** Julien Lie-Panis, Léo Fitouchi, Nicolas Baumard, Jean-Baptiste André

**Affiliations:** ^a^ Department of Brain and Cognitive Sciences, Massachusetts Institute of Technology, Cambridge, MA 02139; ^b^ Department of Psychology, University of Guelph, Guelph, ON N1G 2W1, Canada; ^c^ Max Planck Research Group Dynamics of Social Behavior, Max Planck Institute for Evolutionary Biology, Plön 24306, Germany; ^d^ Department of Social and Behavioral Sciences, Institute for Advanced Studies in Toulouse, Toulouse School of Economics, University of Toulouse Capitole, Toulouse 31080, France; ^e^ Institut Jean Nicod, Département d’études Cognitives, Ecole Normale Supérieure, Université Paris Sciences & Lettres, Ecole des hautes études en sciences sociales, CNRS, 75005 Paris, France

**Keywords:** morality, deontology, cooperation, norms, evolutionary game theory

## Abstract

Humans are capable of remarkably flexible moral judgment. Yet societies rely on rigid rules—obligations and prohibitions that apply categorically, even when case-by-case reasoning could yield better outcomes. Why would a species capable of flexibility bind itself to rigid rules? We propose that rigid rules arise as social technologies for managing ambiguity about noncooperation. People often have legitimate reasons for failing to cooperate, yet those reasons are typically opaque to observers, allowing opportunists to disguise selfishness as justified hardship. We formalize this idea with a game-theoretic model. Two cooperative equilibria emerge: a flexible norm that accommodates legitimate excuses but is vulnerable to exploitation, and a rigid norm that closes this loophole by mandating cooperation even when inefficient. Comparing these equilibria reveals an efficiency–robustness trade-off: Flexibility maximizes welfare when trust is secure, whereas rigidity preserves cooperation when trust is fragile. This explains why rigid rules prevail in interactions with strangers, formal institutions, or in tight societies, while flexibility is more common when trust is secure.

## Significance

Human moral life is governed by rigid rules. Across societies, people rely on categorical prohibitions and duties—do not lie, do not steal, always keep your promises—even when they recognize compelling reasons to make exceptions. Using a mathematical model, we show that rigid rules can protect cooperation when motives are opaque. Flexible rules allow justified exceptions but also make it easier for cheaters to hide behind plausible excuses. This creates a trade-off between maximizing welfare and protecting cooperation from those who would exploit excuses to justify selfishness. Our account explains why rigid rules are common when others are perceived as unreliable, and why many moral judgments feel like nonnegotiable duties rather than cost–benefit calculations.

“Please keep off the grass—unless you have a good reason.” This sign, once posted on the lawn of an Oxford college, represents a flexible approach to collective action. Rather than enforcing a categorical prohibition (“Keep off the grass!”), it invited individuals to exercise judgment, distinguishing between mere convenience (avoiding a short detour) and genuine need (rushing to the hospital). Rigidly staying off the grass would surely keep the lawn immaculate, but at needless cost. By allowing people to weigh the costs and benefits of every detour, the rule achieved its goal efficiently, protecting the grass while permitting legitimate exceptions.

Humans routinely exercise this kind of context-sensitive moral reasoning, adjusting their judgments to the fine-grained parameters of each situation (1–11). When waiting in line, we readily distinguish between someone who cuts out of impatience and someone who cuts because they are diabetic and urgently need sugar (12). This capacity for flexible moral judgment emerges by middle childhood (13–15), suggesting that it is a central feature of human cognition.

Yet few social rules make use of this human flexibility. Across societies—from small-scale foraging bands (16, 17) to large nation-states (18)—cooperation rests on standardized rules that hold regardless of context. In industrialized societies, drivers must obey speed limits and stop at red lights even when roads are empty and conditions are perfectly safe. Laws prohibit stealing others’ property regardless of need, although people readily judge theft to avert starvation as more excusable than theft for greed (19, 20, 21; cf. *Southwark London Borough Council v Williams*^22^). In many subsistence societies, elaborate sharing rules divide a kill by fixed roles—the spotter, the first striker, the harpoon owner—irrespective of who contributed most that day (23–27). Across these settings, rules apply categorically, even when taking context into account could make cooperation more efficient.

This contrast between flexible minds and rigid rules presents a puzzle: Why would a species of flexible cooperators bind itself to categorical rules that demand cooperation even when it is inefficient?

We propose that rigid norms persist because they solve a problem intrinsic to reputation-based cooperation: opacity about why people fail to cooperate.

To illustrate, imagine two roommates, Alice and Bob, trying to divide the household chores. They could adopt a flexible arrangement, letting each clean when they judge, in good faith, that it is their turn. This promises efficiency: There is no point in requiring someone to scrub the bathroom when they are sick or buried in work—the cost to them outweighs the benefit of a cleaner apartment.

But flexibility creates moral wiggle room: ambiguity that an opportunist can exploit (e.g., refs. 28 and 29). If Bob walks into a cluttered kitchen and Alice reports being overwhelmed with work, he cannot tell whether this reflects genuine hardship or a convenient excuse. That opacity shifts Alice’s incentives by giving her cover to exaggerate her workload when she feels lazy. Flexibility thus opens the door to strategic defection, leaving opportunists room to exploit reputational ambiguity.

A rigid rule closes this loophole. Suppose chores are assigned on a fixed schedule—Alice cleans on Sundays and Bob on Thursdays. Failing to clean on one’s scheduled day is then unambiguously a defection. The rigid rule draws a bright line between cooperation and defection, eliminating the ambiguity that opportunists depend on.

We formalize this idea with a model of reputation-based cooperation. In the model, individuals sometimes face genuine hardship—illness, heavy workload, or personal crises—that makes cooperation socially inefficient. Because observers cannot reliably verify these circumstances, actors have room to disguise selfish behavior as justified hardship.

We identify two distinct cooperative equilibria. Under a flexible norm, cooperation accommodates context: individuals who appear to face hardship retain others’ trust. Under a rigid norm, by contrast, cooperation is demanded categorically—any failure to cooperate leads to loss of trust, regardless of apparent circumstances. Comparing these equilibria reveals a fundamental trade-off between efficiency and robustness. Flexibility is efficient, allowing individuals to opt out when cooperation would be genuinely costly, but it leaves observers vulnerable to strategic defection. Rigidity, though occasionally inefficient, makes cooperation more robust, extending it to environments where trust is fragile and flexibility too risky.

In this sense, rigid moral rules act as a social technology: They trade efficiency for robustness, extending the reach of reputation-based cooperation. Based on this model, we predict that flexibility will prevail in high trust environments—such as families, friendships, and tightly knit teams—or when dealing with easily verified hardships like physical injury. Rigid rules, by contrast, should emerge among strangers, in large groups, or in low-trust societies.

More broadly, this logic helps explain a central puzzle in moral psychology: why humans often treat certain rules and values as sacred, insisting on duty regardless of consequence. What appears as deontological rigidity may instead reflect an adaptive response to the problem of sustaining cooperation under uncertainty.

## Model

### Model Overview.

How should observers judge noncooperation when exculpatory circumstances are opaque? Reputation-based cooperation depends on observers being able to infer future behavior from past actions. Yet behavior reflects circumstances as well as character, and circumstances are often private.

We consider a repeated game with two roles (building on ref. 30; for a recent formulation, see ref. 31): signalers, who make cooperative decisions over time and build reputations, and choosers, who each face a single trust decision. This setup separates two adaptive problems. For choosers, the challenge is trust—predicting whether a partner will reciprocate if trusted. For signalers, the challenge is reputation management—bearing the costs of cooperation now to secure trust in the future. Signalers differ in their time horizons. This variation makes cooperative behavior informative about future trustworthiness: More patient signalers are more willing to incur short-term costs to maintain their reputation, whereas less patient signalers are more likely to exploit opportunities for immediate benefit. Without such variation, reputation would carry no information, and cooperation would unravel.

We add ambiguous exculpatory circumstances to this framework. Before interacting with choosers, signalers face a cooperative decision in which some experience genuine hardship that makes cooperation inefficient. Observers cannot reliably verify these circumstances, and those who defect opportunistically can claim hardship as an excuse. This creates the key inference problem for choosers: When a signaler has failed to cooperate but appears to have faced hardship, should they be trusted anyway?

### Players and Basic Structure.

We consider a repeated game with two roles: n≫1 signalers and an infinite pool of choosers. Signalers make cooperative decisions across rounds and hold reputations that reflect their actions. Choosers, in contrast, participate in only one round and decide whether to trust a signaler based on their reputation.

Signalers differ in their time preferences. Each signaler is characterized by a private discount factor δ (with values between 0 and 1), which measures how much they value future payoffs relative to immediate ones; higher δ means greater patience. The distribution of discount factors is common knowledge and assumed only to have full support on (0,1). This produces a spectrum of patience levels, capturing the full range of cooperative tendencies. In particular, it allows for strategic actors who exploit flexible norms but are restrained by rigid ones—the kind of behavior that rigid rules are designed to prevent.

**Fig. 1.**
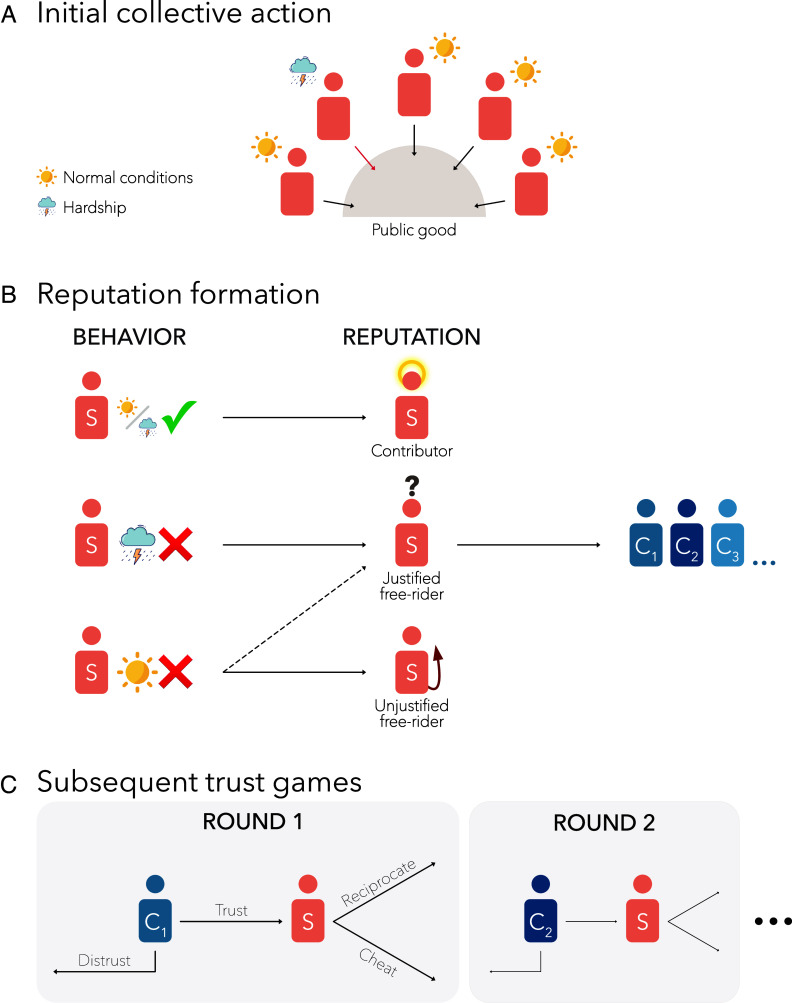
Model overview. (*A*) Initially, signalers decide whether to contribute to a public good. Contribution is efficient under normal conditions but inefficient under hardship. (*B*) Signalers acquire reputations based on their behavior. Those who free-ride under normal conditions are labeled justified free-riders with probability ε, making them indistinguishable from those who free-ride under genuine hardship. The parameter ε captures plausible deniability—how easily opportunists can be mistaken for genuinely hard-pressed individuals. (*C*) Subsequently, signalers interact with choosers, who decide whether to trust based on reputation; if trusted, signalers decide whether to reciprocate.

As illustrated in Fig. 1, the game proceeds in two phases: Signalers first participate in a public goods game, before interacting with choosers in binary trust games.

### Initial Collective Action Under Potential Hardship.

In the initial interaction, signalers decide whether to contribute to a collective action or free-ride on others’ contributions. They play a linear public goods game, where each contribution imposes a private cost on the signaler but generates a public benefit for others.

The cost of contribution depends on circumstances. Under normal conditions, contribution imposes a relatively low private cost γ. However, with probability p, a signaler experiences hardship—illness, competing obligations, or a personal crisis—that increases their cost of contribution to $\gamma_{H}$.

Each contribution yields a public benefit β, which is shared equally among the other signalers (excluding the contributor). The net social value of a contribution is therefore β−γ under normal conditions and $\beta -\gamma_{H}$ under hardship. We assume:

$$0<\gamma <\beta <\gamma_{H},$$

making contributions socially efficient under normal conditions (β−γ>0), but inefficient under hardship ($\beta -\gamma_{H}<0$).

Signalers decide whether to contribute based on their circumstances. Choosers perfectly observe the outcome (contribution or free-riding), but not the underlying circumstances that led to that decision.

As shown in Fig. 1*B*, this partial observability generates reputational ambiguity. Signalers who contribute become contributors, regardless of circumstances. Those who free-ride under hardship become justified free-riders. However, those who free-ride under normal conditions mistakenly become justified free-riders with probability ε, and are otherwise labeled unjustified free-riders.

The parameter ε captures the degree of plausible deniability: How easily opportunistic free-riders can appear genuinely hard-pressed. We model this as observational noise for simplicity, but an equivalent formulation would let individuals who free-ride under normal conditions falsely claim hardship at no cost, succeeding with probability ε—an opportunity they would all take in equilibrium. This assumption preserves the strategic logic of plausible deniability without adding unnecessary complexity.

### Subsequent Trust Games.

After the initial stage, each signaler interacts with a sequence of choosers. In every round, a new chooser decides whether to trust the signaler, incurring cost c to provide benefit b (with 0<c<b). If trusted, the signaler can reciprocate—incurring cost c to return benefit b to the chooser—or cheat, keeping the benefit without giving back. These trust games are one-shot for choosers but repeated for signalers, creating an incentive to maintain a cooperative reputation over time.

After each round, a signaler’s reputation updates to reflect their most recent behavior. The full reputation combines their initial collective action label (contributor, justified free-rider, or unjustified free-rider) with their most recent behavior in trust games (reciprocator or cheater), if any. Choosers rely on this composite reputation when deciding whether to place their trust.

Our two-stage structure resembles that of Panchanathan and Boyd (32), who also study a public goods game followed by repeated dyadic interactions. We depart by adopting a signaling lens and introducing opaque exculpatory circumstances. Signalers are long-run players with different time preferences that shape their willingness to cooperate for reputational benefits, while choosers are short-run players who use reputation to predict cooperativeness. Hardship with plausible deniability in the initial stage creates the reputational ambiguity that is central to our analysis. The specific form of the initial interaction is not essential: Any cooperative decision under opaque circumstances would generate the same structure.

## Results

The analysis reveals two cooperative equilibria, Perfect Bayesian Equilibria that sustain reputation-based cooperation (see Methods for a formal definition): a flexible norm that accommodates apparent hardship, and a rigid norm that categorically demands cooperation regardless of context.

For clarity, we assume:

$$\frac{\gamma }{1-\varepsilon }<c<\gamma_{H}.$$

This assumption ensures that contributing under normal conditions is less demanding than reciprocating trust (left inequality), and that contributing under hardship is more demanding (right inequality). It simplifies the behavioral thresholds below without changing any qualitative result (see *SI Appendix* for the general case).

### Two Equilibria: Flexible and Rigid Norms.

#### Flexible norm.

In this equilibrium, choosers interpret free-riding leniently: If a signaler appears to have faced hardship, they are still deemed trustworthy. In the initial trust game, before a signaler’s reciprocation behavior is known, trust is extended to contributors and justified free-riders, but withheld from unjustified free-riders. Later, once reciprocation is observed, choosers trust reciprocators and distrust cheaters.

Signalers never contribute under hardship, since free-riding then carries no reputational penalty. Under normal circumstances, however, they face a trade-off: Free-riding avoids the immediate cost of contribution, γ, but risks losing the next chooser’s trust—worth b—with probability 1−ε, if the signaler’s true circumstances are observed. Signalers then contribute if and only if their discount factor satisfies $\delta \geq \delta_{\text{normal}}^{\text{flex.}}$, where:

[F1] $$\begin{array}{c} \delta_{\text{normal}}^{\text{flex.}}=\frac{\gamma }{(1-\varepsilon )b}. \end{array}$$

In subsequent trust games, reciprocation incurs cost c but secures next chooser’s trust, worth b. Signalers reciprocate if and only if their discount factor satisfies $\delta \geq \delta_{\text{recip.}}$, where:

$$\delta_{\text{recip.}}=\frac{c}{b}.$$

Because $\frac{\gamma }{1-\varepsilon }<c$, this threshold is higher than $\delta_{\text{normal}}^{\text{flex.}}$; contributing under normal circumstances requires less patience than reciprocating trust.

From these thresholds, three signaler types emerge. Impatient signalers ($\delta <\delta_{\text{normal}}^{\text{flex.}}$) always free-ride and always cheat, regardless of circumstances or reputation. Intermediate signalers ($\delta_{\text{normal}}^{\text{flex.}}\leq \delta <\delta_{\text{recip.}}$) contribute under normal circumstances but cheat when trusted. Patient signalers ($\delta \geq \delta_{\text{recip.}}$) contribute under normal circumstances and always reciprocate trust.

The flexible norm is sustainable whenever initial trust decisions are well founded. The binding constraint involves justified free-riders, who must reciprocate frequently enough to make trusting them worthwhile. The flexible norm exists if and only if:

[F2] $$\begin{array}{c} \frac{p\;P(\delta \geq \delta_{\text{recip.}})}{p+\left(1-p\right)\varepsilon \;P\left(\delta <\delta_{\text{normal}}^{\text{flex.}}\right)}\geq \frac{c}{b}. \end{array}$$

This condition ensures that justified free-riders are, on average, sufficiently likely to reciprocate relative to the cost-to-benefit ratio of trust, $\frac{c}{b}$. The numerator gives the probability that a reciprocator acquires the justified free-rider reputation—necessarily by facing hardship—while the denominator gives the overall probability of that reputation: All signalers become justified free-riders when they face hardship, with probability p, and free-riders also acquire this reputation when they face normal conditions and successfully misrepresent them as hardship, with probability (1−p)ε.

#### Rigid norm.

In this equilibrium, choosers adopt a zero-tolerance policy: Only contributors are trusted, while any instance of free-riding—regardless of apparent circumstances—results in loss of trust. In the initial trust game, only contributors are trusted, while both justified and unjustified free-riders are distrusted. Later, choosers trust reciprocators and distrust cheaters.

Signalers now face stronger incentives to contribute under normal conditions. They do so if and only if their discount factor satisfies $\delta \geq \delta_{\text{normal}}^{\text{rigid}}$, where:

[R1] $$\begin{array}{c} \delta_{\text{normal}}^{\text{rigid}}=\frac{\gamma }{b}. \end{array}$$

This threshold is lower than under the flexible norm ($\frac{\gamma }{b}<\frac{\gamma }{b(1-\varepsilon )}$). Signalers whose discount factor falls between these two thresholds can be understood as opportunists: They contribute when rigidity prevents misrepresentation but free-ride when flexibility allows reputational ambiguity to be exploited—opportunists, in short, exploit moral wiggle room when it is available.

The rigid norm also creates incentives to contribute under hardship. A signaler facing hardship contributes if and only if their discount factor satisfies $\delta \geq \delta_{\text{hardship}}^{\text{rigid}}$, where:

[R2] $$\begin{array}{c} \delta_{\text{hardship}}^{\text{rigid}}=\frac{\gamma_{H}}{\gamma_{H}+b-c}. \end{array}$$

Signalers reciprocate trust if and only if their discount factor satisfies $\delta \geq \delta_{\text{recip.}}=\frac{c}{b}$, as with the flexible norm. From these thresholds, four types of signalers emerge. Very impatient signalers ($\delta <\delta_{\text{normal}}^{\text{rigid}}$) always free-ride and cheat; impatient signalers ($\delta_{\text{normal}}^{\text{rigid}}\leq \delta <\delta_{\text{recip.}}$) contribute under normal circumstances but cheat when trusted; patient signalers ($\delta_{\text{recip.}}\leq \delta <\delta_{\text{hardship}}^{\text{rigid}}$) contribute under normal circumstances and reciprocate; and very patient signalers ($\delta \geq \delta_{\text{hardship}}^{\text{rigid}}$) contribute even under hardship and reciprocate.

The rigid norm is sustainable whenever initial trust decisions are well founded. There are two binding conditions, involving contributors and justified free-riders respectively. The rigid norm exists if and only if:

[R3] $$\begin{array}{c} \frac{p\;P(\delta_{\text{recip.}}\leq \delta <\delta_{\text{hardship}}^{\text{rigid}})}{p\;P\left(\delta <\delta_{\text{hardship}}^{\text{rigid}}\right)+\left(1-p\right)\varepsilon \;P\left(\delta <\delta_{\text{normal}}^{\text{rigid}}\right)}<\frac{c}{b}, \end{array}$$

[R4] $$\begin{array}{c} \frac{\left(1-p\right)\;P\left(\delta \geq \delta_{\text{recip.}}\right)+p\;P\left(\delta \geq \delta_{\text{hardship}}^{\text{rigid}}\right)}{\left(1-p\right)\;P\left(\delta \geq \delta_{\text{normal}}^{\text{rigid}}\right)+p\;P\left(\delta \geq \delta_{\text{hardship}}^{\text{rigid}}\right)}\geq \frac{c}{b}. \end{array}$$

Condition [R3] ensures that justified free-riders are sufficiently unlikely to reciprocate, making it sensible to distrust them, while condition [R4] ensures that contributors are sufficiently likely to reciprocate, making trust worthwhile. In both cases, the numerators give the probability that a reciprocator holds the relevant reputation, and the denominators give its overall probability in the population.

### Comparing Flexible and Rigid Norms.

These two equilibria represent distinct approaches to moral judgment under uncertainty. The flexible norm extends trust to those who appear to have faced hardship; the rigid norm withholds it. As captured by conditions [F2 and **5**], whether such trust is well founded depends on how likely these justified free-riders are to reciprocate trust.

That likelihood, in turn, depends on two features of the environment: the distribution of discount factors, which shapes the population’s cooperativeness, and plausible deniability, which measures how easily selfish behavior can be disguised as hardship.

**Fig. 2.**
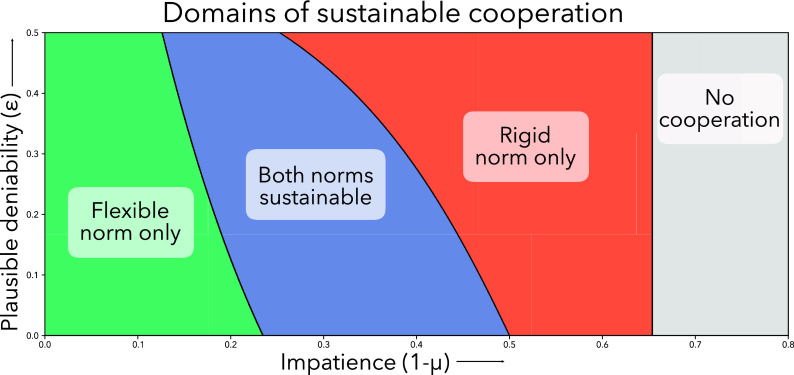
Domains of sustainable cooperation. Each point represents a social environment defined by impatience 1−μ (x-axis) and plausible deniability ε (y-axis). Colors indicate where the flexible [F2] and rigid [R3 and R4] norms are sustainable. Green: Only the flexible norm is sustainable; orange: only the rigid norm; blue: both; white: Cooperation fails. Discount factors follow a normal distribution with mean μ and SD σ=0.3, truncated to the interval (0,1). Parameters: $c=1,b=2,\gamma =0.5,\beta =0.75,\gamma_{H}=2,$ and p=0.2. We vary impatience 1−μ between 0 and 0.75 (i.e., μ between 1 and 0.25) and plausible deniability ε between 0 and $1-\frac{\gamma }{c}=0.5$, ensuring that γ/(1−ε)<c holds throughout.

To illustrate, we assume signalers’ discount factors follow a normal distribution with mean μ and SD σ, truncated to the interval (0,1). Fig. 2 shows the resulting domains of sustainable cooperation as functions of impatience (1−μ) and plausible deniability (ε). When the population is patient and deniability is low, only the flexible norm is sustainable: Trust can safely extend to justified free-riders, who are likely to reciprocate and to have faced genuine hardship. As impatience and deniability increase, the flexible norm collapses, and cooperation can persist only under the rigid norm. When impatience becomes extreme, even contributors can no longer be trusted, and cooperation breaks down altogether.

**Fig. 3.**
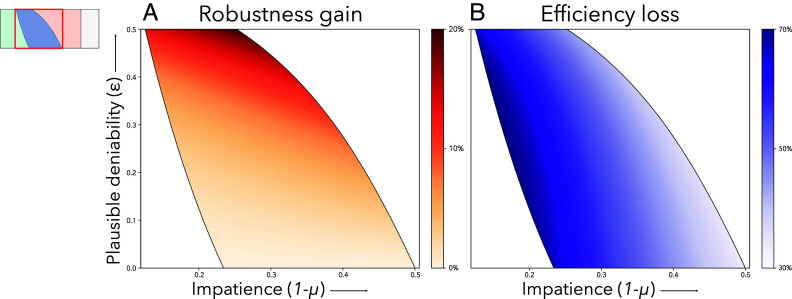
The efficiency–robustness trade-off. Each point represents a social environment defined by impatience 1−μ (x-axis) and plausible deniability ε (y-axis). We restrict attention to the domain where both norms are sustainable (blue region in Fig. 2) and compare their performance. (*A*) Robustness gain from adopting the rigid norm. Warm shades (yellow to red) show the fraction of opportunists who, under normal conditions, would free-ride under the flexible norm but contribute when cooperation is categorically required. (*B*) Efficiency loss from adopting the rigid norm. Purple shades show the normalized payoff difference from the initial collective action (flexible–rigid). Parameters and distribution of discount factors as in Fig. 2.

Rigid norms do more than offer an alternative to flexible ones—they enable cooperation in contexts where flexibility would fail. Fig. 3 shows how rigid norms achieve this by comparing performance where both norms are possible. The rigid norm is more robust to strategic defection but less efficient: It prevents opportunistic exploitation of ambiguity (Fig. 3*A*), but incentivizes cooperation under hardship, leading to an overall payoff loss (Fig. 3*B*).

Thus, rigid norms function as social technologies that trade efficiency for robustness against strategic defection, extending the domain of cooperation by eliminating exploitable ambiguity.

### Generality Beyond Cooperative Signals.

Signalers initially face a cooperative decision: whether to contribute to a collective action that generates a shared benefit (β>0). Patient signalers are more likely to initially contribute and later reciprocate trust, so choosers can infer trustworthiness from behavior in the collective action. The initial interaction also has ambiguous exculpatory circumstances, allowing us to answer our central question: When do rigid versus flexible rules for judging cooperative behavior emerge?

Note that the public benefit of contribution, β, does not appear in any equilibrium condition. Contributions are sustained because they allow signalers to secure choosers’ trust in subsequent interactions, not because of their direct returns in the initial stage. Contributions secure trust when they serve as sufficiently good signals of propensity to incur the cost of reciprocation (c), which, in turn, depends on the propensity to incur the relevant cost of contribution (γ or $\gamma_{H}$)—but not on the benefit it creates (β). Our results thus extend beyond the cooperative domain: they hold even when contributions provide no social benefit (β=0), as with certain loyalty signals. When loyalty is uncertain, observers may demand costly displays even from those who claim legitimate excuses. We analyze this case in *SI Appendix*, section 3.

## Discussion

### Rigid Rules As Social Technologies.

Why do societies adopt rigid moral rules when humans are perfectly capable of flexible, context-sensitive moral reasoning? Our model addresses this question by examining how cooperation evolves when people’s circumstances are private and reputations ambiguous.

Individuals in our model sometimes face genuine hardship—situations in which cooperating would be socially inefficient. Yet observers cannot perfectly assess these circumstances: With some probability, an individual who defects under normal conditions is mistaken for someone acting under hardship. This probability captures the degree of plausible deniability—how easily selfish behavior can appear as genuine constraint. Individuals also differ in their time preferences: More future-oriented individuals are more willing to incur the immediate costs of cooperation to maintain their reputation, whereas present-oriented individuals prioritize short-term gains.

The analysis reveals two cooperative equilibria, representing two approaches to moral judgment under uncertainty: a flexible norm that accommodates apparent hardship, and a rigid norm that categorically demands cooperation regardless of context. These equilibria succeed under different conditions. The flexible norm is sustainable when trust is secure; when plausible deniability is low and individuals are future-oriented, those who appear hard-pressed are genuinely trustworthy, and contextual discretion does not invite exploitation. In contrast, when individuals are present-oriented and hardship is easy to fake, flexible trust becomes too risky, but cooperation can be sustained under the rigid norm.

The rigid norm thus extends cooperation to settings where flexible trust would fail. Comparing it to the flexible norm reveals an efficiency–robustness trade-off: By treating all defections alike, rigidity makes trust decisions robust to strategic defection, but at the cost of sometimes enforcing inefficient cooperation. Rigid norms appear as social technologies that trade efficiency for robustness.

### Empirical Predictions.

Our model predicts that rigid rules—those that demand cooperation even in apparent hardship—should arise when flexibility is most risky; that is, when the potential for strategic defection is highest.

Two factors drive this risk. The first is the opacity of exculpatory circumstances, captured by the parameter ε in our model. Consider pregnancy: Women in late stages, where their condition is more visible, often receive accommodation, such as seats on public transport or flexibility at work. Such accommodations are much rarer in early pregnancy, when needs are comparable but less apparent. More generally, hard-to-verify claims like stress or fatigue should receive less accommodation than observable injuries.

The second factor is the perceived reliability of cooperation partners. In our model, reliability is driven by time preferences: More patient signalers (higher δ) cooperate more broadly (consistent with empirical evidence; e.g., refs. 33 and 34), including when they could exploit moral wiggle room. As a result, flexible norms are easier to sustain when the population of signalers is more patient (higher μ)—when choosers have reason to believe that their partners are patient and therefore reliable. The following predictions derive from variation in perceived patience across individuals, relationships, and societies.

We first predict that moral rules should be applied more rigidly to individuals perceived as impulsive or untrustworthy. This may explain why young children are taught categorical moral rules with little room for contextual nuance—they are not yet seen as reliable enough to exercise moral discretion (see also ref. 13). A similar logic shapes attitudes toward welfare: When recipients are perceived as lazy rather than unlucky, people tend to favor strict eligibility rules (35).

Second, we predict that rigid rules should be more frequent in relationships with shorter time horizons. Flexibility should be more common when the shadow of the future looms large, such as with family and friends, but rigid rules should dominate in more transient relationships, such as between colleagues or strangers. This sheds light on relational psychology (36). Recent work suggests that formality is a fundamental dimension of relational psychology, distinguishing public relationships that adhere to rules and regulations from private relationships with looser, more casual styles (37). Consistent with this prediction, relationships become more formal in modernized societies, where interactions increasingly involve strangers rather than intimate social circles.

Third, societies where time horizons are shorter should adopt more rigid rules. This helps explain why some societies are tight—with many strong norms and a low tolerance of deviant behavior—and others are loose (18). Tightness is associated with ecological and social threats, such as disease, natural disasters, resource scarcity, population density, and intergroup conflict (38–40). Existing accounts attribute this pattern to coordination demands: groups under threat require stronger norms to sustain collective action (formalized in ref. 41). Our model complements this view by identifying a cognitive mechanism that makes rigidity appealing. In threatening environments, immediate needs loom larger and future benefits are more uncertain, shortening time horizons (42–44), and making investment in one’s cooperative reputation less worthwhile. Recognizing this, people expect others to be less reliably cooperative and see flexibility as too risky, turning instead to more rigid rules—the loss in efficiency is outweighed by the gain in robustness. As shown by Nettle and Saxe (45), a similar mechanism underlies support for authoritarian leaders and punitive regulation, explaining why these political preferences also rise under threat (46–49).

### The Logic of Moral Rigidity.

Moral judgments are often strikingly deontological: People treat certain rules, values, and rights as sacred or inalienable, refusing to weigh them against costs, benefits, or pragmatic compromises (1, 50–53). One way to understand this rigidity is through the efficiency–robustness trade-off. As we have shown, categorical rules close loopholes, making cooperation easier to sustain. The same logic extends more broadly: Rigid principles keep expectations clear, make departures easier to detect or sanction, and prevent minor allowances from hardening into precedents that legitimize further slippage, gradually weakening the cooperative order (54). People may therefore endorse some rules categorically not because they view them as intrinsically right, but because they sense that strict adherence is the most reliable way to sustain cooperation in the long run.

Our account complements existing explanations for moral rigidity. Rules can act as cognitive shortcuts (55), helping individuals approximate–and coordinate on–mutually beneficial bargains (56). Bright-line norms can also facilitate coordination among third-party enforcers (57) and bystanders (58). And principled, trade-off–insensitive behavior can serve as a signal of commitment, helping individuals build trust (59, 60).

### Implications for Evolutionary Game Theory.

Our model differs from the standard framework for reputation-based cooperation in three ways.

First, we introduce heterogeneity in cooperative types. In standard indirect reciprocity models, individuals are identical and interactions are repeated (61–63). Norms—understood as rules for assigning good or bad reputation—are then evaluated by their capacity to generate and stabilize cooperation (64–68). We instead assume that signalers vary in patience, integrating the logic of signaling with that of repeated cooperation (building on ref. 30; see also refs. 69 and 70). Patient signalers reciprocate trust to attract the trust of future partners, whereas impatient ones cheat. Different norms then emerge depending on which reputations predict reciprocation and which predict cheating. This avoids the scoring dilemma: Cooperation in the trust game is sustainable because first-order reputations (reciprocator or cheater) perfectly predict future behavior.

Second, we add a public goods game, following the structure of Panchanathan and Boyd (32). The signaling framework clarifies when contributions can be stabilized: Signalers must be sufficiently patient for choosers to infer trustworthiness from contribution.

Third, we introduce opaque exculpatory circumstances, a feature rarely modeled (although see ref. 71). This creates opportunities for strategic misrepresentation, leading to reputational ambiguity. Flexible assessments emerge when choosers can trust despite this ambiguity; rigidity becomes necessary when trust is too risky. Adopting a signaling perspective thus reframes norm evolution: stricter assessment rules—akin to Stern Judging in indirect reciprocity models (68, 72–74)—arise as adaptive responses to observers’ inferential challenges.

### Limits and Future Directions.

Our model isolates one adaptive problem—judging trustworthiness under opaque circumstances—and shows how it can produce both flexible and rigid assessments. To do so, we abstract away from features of real interactions that would otherwise obscure this problem: Ambiguity is confined to a single initial collective action, this collective action does not recur, and choosers and signalers occupy distinct roles meant to capture distinct adaptive problems. These assumptions do not match any particular real-world interaction. Their value is theoretical: By isolating one inferential challenge, the model reveals how flexible and rigid rules can each emerge as adaptive responses to it.

What’s more, moral norms are not just solutions to individual inference problems. They involve widely shared expectations about how people ought to behave, the felt obligation to conform, and culturally inherited conventions that come to seem external or binding (75). Real-world cooperation also requires forms of coordination that our analysis sets aside—coordination on the standards we use to assess others, on when and how to enforce them, and on the shared courses of action that make collective endeavors possible. Integrating our approach with models that have examined such dynamics (76–81) is a natural direction for future work. Other extensions include allowing reputational ambiguity or collective action to recur, and complementing our equilibrium analysis with evolutionary dynamics.

## Conclusion

Flexible minds, rigid rules: Our model shows this contrast arises from a simple informational constraint–people often cannot tell whether noncooperation reflects genuine hardship or convenient excuses. This ambiguity gives opportunists room to exploit flexible rules. Rigid rules close that loophole, making cooperation more robust to strategic defection, even at the cost of sometimes enforcing inefficient behavior. This efficiency–robustness trade-off explains when societies tighten norms and why consequentialist moral reasoning can nonetheless give rise to judgments that seem deontological.

## Materials and Methods

### Model Description.

#### Players.

We consider a repeated game with n≫1 long-run signalers and an infinite pool of short-run choosers. Before play begins, signalers are assigned a discount factor δ according to a distribution with full support on Δ=(0,1). They privately observe their discount factor; the distribution is common knowledge.

#### Interactions.

The game proceeds in two stages. Initially (round 0), signalers decide whether to contribute to a public good. Each contribution generates collective benefit β, divided equally among the n−1 other signalers. The cost of contribution depends on circumstances: $\gamma_{H}$ under hardship (probability p∈(0,1)) and γ under normal conditions (probability 1−p), where $0<\gamma <\beta <\gamma_{H}$. Signalers privately observe their cost before deciding.

Subsequently (rounds t≥1), each signaler plays a repeated trust game with a sequence of choosers. In every round, the signaler is paired with a new chooser, who decides whether to trust them, incurring cost c to provide benefit b (where 0<c<b). If trusted, the signaler chooses whether to reciprocate, incurring cost c to return benefit b.

#### Reputations.

Signalers acquire a public goods game reputation $\omega_{\text{pgg}}$ and a trust game reputation $\omega_{\text{tg}}$. Their full reputation is $\omega =(\omega_{\text{pgg}},\omega_{\text{tg}})\in \Omega$.

There are three possible values for $\omega_{\text{pgg}}$. Signalers who contribute become contributors, those who free-ride under hardship become justified free-riders, and those who free-ride under normal conditions become justified free-riders with probability ε∈(0,1) and unjustified free-riders with probability 1−ε.

There are three possible values for $\omega_{\text{tg}}$. Signalers who have not yet been trusted have unobserved trust game behavior; those who last reciprocated are reciprocators; and those who last cheated are cheaters.

For simplicity of exposition, when a signaler’s trust game behavior is unobserved ($\omega_{\text{tg}}=\;\text{unobserved}$), we refer to them by their public goods game reputation alone—for example, contributors are signalers with reputation (contributor,unobserved). Once a signaler has been trusted, we refer to them by their trust game reputation—for example, reciprocators are signalers with any reputation (·,reciprocator).

#### Strategies and beliefs.

A signaler public goods game strategy, represented by a function $\sigma_{\text{pgg}}:\Delta \times \left\{\text{normal},\;\text{hardship}\right\}\to \left\{\text{contribute},\;\text{free-ride}\right\}$, specifies whether to contribute depending on discount factor and circumstances. A signaler trust game strategy, represented by a function $\sigma_{\text{tg}}:\Delta \times \Omega \to \left\{\text{reciprocate},\;\text{cheat}\right\}$, specifies whether to reciprocate trust depending on discount factor and reputation.

Choosers hold shared beliefs ϕ(·∣ω) over Δ for each reputation ω∈Ω, encoding how they assign likelihood to different signaler discount factors, conditional on observed reputation. A chooser strategy, represented by a function $\sigma_{\text{ch}}:\Omega \to \left\{\text{trust},\;\text{distrust}\right\}$, specifies whether to trust a signaler based on their reputation.

A strategy profile is $\sigma =(\sigma_{\text{pgg}},\sigma_{\text{tg}},\sigma_{\text{ch}})$.

### Equilibrium Analysis.

#### Equilibrium concept.

We study the model’s Perfect Bayesian Equilibria (PBEs) in pure strategies. A PBE is a pair (σ,ϕ) where players have no profitable deviations at any history (on or off the equilibrium path), and beliefs are updated according to Bayes’ rule whenever possible (82). PBE is the standard solution concept for sequential games with incomplete information, and agrees with evolutionary simulations in related settings (31, 83).

#### Cooperative equilibria.

We focus on cooperative PBEs, in which choosers’ trust decisions incentivize cooperation at every history. Choosers must trust contributors and distrust unjustified free-riders (incentivizing contributions to the public good), and trust reciprocators and distrust cheaters (incentivizing reciprocation in every subgame). We obtain two cooperative PBEs: the flexible and the rigid norm.

In *SI Appendix*, we characterize the model’s other PBEs. These fall into two categories: PBEs that are outcome-equivalent to the flexible or rigid norm, differing only in off-path play; and PBEs in which signalers never contribute to the public good.

### Numerical Solution.

#### Evaluation of domains and performance.

We numerically evaluate the domain conditions for the flexible [**2**] and rigid [R3–**6**] norms on a parameter grid. These conditions are stated in terms of probabilities over discount factors; to evaluate them, we fix the distribution of discount factors to a normal with mean μ and SD σ=0.3, truncated to (0,1) (so μ refers to the pretruncation mean). We vary impatience 1−μ and plausible deniability ε, holding other parameters fixed as in the figure captions. Fig. 2 displays the domains of sustainable cooperation by identifying parameter combinations for which these conditions hold; Fig. 3 compares performance where both norms are sustainable.

## Supplementary Material

Appendix 01 (PDF)

## Data Availability

No new empirical data were generated for this study. All analytical derivations are provided in *SI Appendix*. The code used to generate Figs. 2 and 3 is available in the public OSF repository for this paper (https://osf.io/2jxry) (84).
