# Supplementary material for: Why human societies adopt rigid moral rules: The efficiency–robustness trade-off
Source: Proc Natl Acad Sci U S A. 2026 Jul 8;123(28):e2535467123. doi: 10.1073/pnas.2535467123 (PMC13367805; doi:10.1073/pnas.2535467123)
Supplement: Supplementary file 1 — Appendix 01 (PDF) [file pnas.2535467123.sapp.pdf]

Supplementary Information

Why human societies adopt rigid moral rules: the efficiency–robustness trade-off

Julien Lie-Panis[1], Léo Fitouchi[2], Nicolas Baumard[3] and Jean-Baptiste André[3]

[1] Max Planck Institute for Evolutionary Biology
[2] Institute for Advanced Studies in Toulouse
[3] Institut Jean Nicod

Contents

Summary **2**

1 Model **2**
1.1 Players . . . 2
1.2 Stage 1: Linear public goods game with potential hardship . . . 3
1.3 Stage 2: Repeated trust games . . . 3
1.4 Reputations . . . 3
1.5 Strategies and beliefs . . . 4
1.6 Payoffs . . . 4
1.7 Equilibrium concept . . . 5

2 Cooperative equilibria **6**
2.1 Flexible norm . . . 6
2.2 Rigid norm . . . 7
2.3 Other equilibria . . . 8

3 Beyond cooperative signals **10**
3.1 β does not enter the equilibrium conditions . . . 10
3.2 Welfare comparison when $\beta = 0$ . . . 10

A Demonstrations for the cooperative equilibria **11**
A.1 Preliminary: continuation payoffs . . . 11
A.2 Trust game behavior . . . 12
A.3 Flexible norm . . . 13
A.4 Rigid norm . . . 17

B Demonstrations for other equilibria **22**
B.1 General results . . . 23
B.2 Equilibria with initial contributions . . . 24
B.3 Equilibria without initial contributions . . . 25

List of Figures

1 Key parameters . . . 2
2 Comparison between cooperative and money-burning signals . . . 11
3 Signaler behavior under the flexible norm . . . 15
4 Signaler behavior under the rigid norm . . . 20

Overview

This Supplementary Information presents the formal details and proofs supporting the main text. Section 1 defines the complete game-theoretic model, including the players, the two-stage game structure (a public goods game followed by repeated trust games), the formation of reputations, and the equilibrium concept used. Section 2 characterizes the two cooperative equilibria—the flexible norm that accommodates claims of hardship and the rigid norm that categorically demands contribution to the public good—and derives their conditions for existence. Appendix A provides detailed proofs for these cooperative equilibria, while Appendix B characterizes other possible equilibria, showing that all equilibria with public good contributions are outcome-equivalent to either the flexible or the rigid norm. Key notation and parameters are summarized in the table below.

**Public goods game**	γ	Cost of contribution under normal conditions
	γ_H	Cost of contribution under hardship
	β	Public benefit from contribution
	p	Probability of hardship
	ε	Plausible deniability
**Trust games**	c	Cost of trust and reciprocation
	b	Benefit of receiving trust or reciprocation
**Types and thresholds**	δ	Signaler's discount factor
	$\delta_{\text{recip.}}$	Reciprocation threshold
	$\delta^{\text{flex.}}_{\text{normal}}$	Contribution threshold (flexible norm)
	$\delta^{\text{rigid}}_{\text{normal}}$	Contribution threshold (rigid norm)
	$\delta^{\text{rigid}}_{\text{hardship}}$	Hardship contribution threshold (rigid norm)
**Distributions of types (for the numerical solution)**	μ	Pre-truncation mean
	σ	Pre-truncation standard deviation

Supplementary Figure 1: Key parameters and behavioral thresholds in the model, and for the numerical solution used in Figures 2-3 and Supplementary Figure 2.

1 Model

We define the complete game-theoretic model, including the players, stages, reputations, strategies, payoffs, and the equilibrium concept used. In particular, we introduce the notion of cooperative equilibrium—the subset of Perfect Bayesian Equilibria that incentivize cooperation at every history—which provides the foundation for the analysis in Section 2.

1.1 Players

We consider a repeated game with two roles: $n \gg 1$ signalers and infinitely many choosers. Signalers are long-lived: they make cooperative decisions throughout the game and acquire reputations based on those decisions. Choosers are short-lived: each appears in a single round of the repeated game and must decide whether to trust a signaler based on their reputation.

Each signaler is characterized by a discount factor, or type, $\delta \in (0,1)$, which measures how much they value future payoffs relative to immediate ones. At the start of the game, every signaler privately observes their own δ, drawn from a distribution with full support on $\Delta \equiv (0,1)$.

1.2 Stage 1: Linear public goods game with potential hardship

In the initial round of the repeated game ($t = 0$), signalers decide whether to contribute to a public good. Each contribution generates a collective benefit of β, which is evenly divided among the $n-1$ other signalers (excluding the contributor).

The cost of contribution depends on circumstances. With probability p, a signaler faces hardship and a high cost γ_H; with probability $1-p$, conditions are normal and the cost is γ. We assume

$$0 < \gamma < \beta < \gamma_H,$$

so that contributing under normal conditions is socially efficient ($\beta - \gamma > 0$), while contributing under hardship is inefficient ($\beta - \gamma_H < 0$). Signalers privately observe whether they face hardship before deciding whether to contribute.

1.3 Stage 2: Repeated trust games

Subsequently, each signaler plays a repeated trust game with a sequence of choosers. In every round $t \geq 1$, the signaler is paired with a new chooser. The chooser decides whether to trust them, incurring cost c to provide benefit b to the signaler, where $0 < c < b$. If trusted, the signaler decides whether to reciprocate—incurring cost c to provide benefit b to the chooser in return for their trust—or to cheat.

1.4 Reputations

In the public goods game, choosers observe signalers' actions but not the circumstances behind free-riding. Each signaler acquires a public goods game reputation ω_{pgg} based on their observed behavior:

- Signalers who contribute become contributors, regardless of circumstances;
- Signalers who free-ride under hardship become justified free-riders;
- Signalers who free-ride under normal conditions become justified free-riders with probability ε, and are otherwise labeled unjustified free-riders.

The parameter ε captures the degree of plausible deniability: how easily opportunistic free-riders can be mistaken for genuinely hard-pressed individuals.

In trust games, choosers observe only signalers' most recent action. After each round, a signaler's trust game reputation ω_{tg} updates based on their last behavior:

- Signalers who have not yet been trusted have unobserved trust game behavior;
- Signalers who last reciprocated are reciprocators;
- Signalers who last cheated are cheaters.

A signaler's full reputation, $\omega \equiv (\omega_{\text{pgg}}, \omega_{\text{tg}})$, combines both components, and belongs to the set

$$\Omega \equiv \{\text{contributor}, \text{justified free-rider}, \text{unjustified free-rider}\} \times \{\text{unobserved}, \text{reciprocator}, \text{cheater}\}.$$

For example, a signaler who initially faces hardship and opts to free-ride enters round 1 with reputation (justified free-rider, unobserved). Later, if they are trusted and reciprocate, the signaler acquires reputation (justified free-rider, reciprocator). They keep this reputation as long as they reciprocate every time they are trusted; otherwise, after cheating, they acquire the reputation (justified free-rider, cheater).

For simplicity of exposition, when a signaler's trust game behavior is unobserved ($\omega_{\text{tg}} = \text{unobserved}$), we refer to them by their public goods game reputation alone—for example, contributors are signalers with reputation (contributor, unobserved). Once a signaler has been trusted, we refer to them by their trust game reputation when context is clear—for example, reciprocators are signalers with any reputation ($\cdot$, reciprocator). When precision is needed, we use the full notation $(\omega_{\text{pgg}}, \omega_{\text{tg}})$.

1.5 Strategies and beliefs

A signaler strategy for the public goods game is a function

$$\sigma_{\text{pgg}} : \Delta \times \{\text{normal}, \text{hardship}\} \to \{\text{contribute}, \text{free-ride}\},$$

specifying whether to contribute or free-ride depending on type and circumstances.

A signaler strategy for the repeated trust game is a function

$$\sigma_{\text{tg}} : \Delta \times \Omega \to \{\text{reciprocate}, \text{cheat}\},$$

specifying whether to reciprocate a chooser's trust depending on type and reputation.

A chooser strategy is a function

$$\sigma_{\text{ch}} : \Omega \to \{\text{trust}, \text{distrust}\},$$

specifying whether to trust a signaler based on their reputation.

Together, these functions form a strategy profile

$$\sigma \equiv (\sigma_{\text{pgg}}, \sigma_{\text{tg}}, \sigma_{\text{ch}}).$$

For each reputation $\omega \in \Omega$, choosers hold a posterior belief about the types of signalers who carry that reputation. Formally, for every ω,

$$\phi(\cdot \mid \omega)$$

denotes a probability distribution over types $\delta \in \Delta$, conditional on reputation ω. We denote by ϕ the collection of posterior beliefs across all reputations.

1.6 Payoffs

Given a strategy profile σ and beliefs ϕ, we define payoffs for both roles.

A chooser who distrusts a signaler of reputation ω obtains payoff

$$u_{\text{ch}}(\text{distrust} \mid \omega) \equiv 0.$$

If the chooser instead trusts, they pay cost c and receive benefit b if the signaler reciprocates. Their expected payoff is

$$u_{\text{ch}}(\text{trust} \mid \omega) \equiv -c + \mathbb{P}(\text{reciprocate} \mid \omega)\, b,$$

where $\mathbb{P}(\text{reciprocate} \mid \omega)$ is determined by the trust game strategy σ_{tg} together with choosers' posterior beliefs $\phi(\cdot \mid \omega)$. For instance, if choosers believe that signalers with reputation ω tend to have a low discount factor, and if such impatient signalers always cheat according to σ_{tg}, then this probability is 0. For concision, we omit σ_{tg} and $\phi(\cdot \mid \omega)$ from the notation.

Signalers, in contrast, receive payoffs across all rounds of the game, discounted according to their type δ. These payoffs are normalized by the factor $(1-\delta)$, ensuring they remain comparable across types (without this normalization, payoffs would grow arbitrarily large as δ approaches 1). The payoff of a signaler of type δ is

$$U_{\text{sgl}}(\sigma \mid \delta) \equiv (1-\delta)\left(\pi^0_{\text{sgl}} + \sum_{t=1}^{\infty} \delta^t \pi^t_{\text{sgl}}\right),$$

where π^0_{sgl} is the payoff in the public goods game (depending on the contribution decisions of all signalers), and π^t_{sgl} is the payoff in the trust game in round t (depending on the actions induced by σ for the signaler and the assigned chooser).

For simplicity of notation, we assume that players choose the cooperative action when indifferent between it and its uncooperative alternative: signalers contribute when indifferent between contributing and free-riding, they reciprocate when indifferent between reciprocating and cheating, and choosers trust when indifferent between trusting and distrusting. These indifference cases occur only at knife-edge parameter values.

1.7 Equilibrium concept

We limit our analysis to Perfect Bayesian Equilibria (PBE) in pure strategies. Following Fudenberg and Tirole [1, Chapter 8], a PBE is a pair (σ, ϕ), where σ is a strategy profile and ϕ a system of beliefs, such that players have no profitable deviations in any possible scenario (on or off the equilibrium path), and beliefs are updated according to Bayes' rule whenever possible.

This requires considering all potential deviations. For signalers, we must verify that no type δ has an incentive to deviate from σ_{pgg} in the public goods game, under either hardship or normal conditions. Likewise, no signaler type δ with any reputation ω should have an incentive to deviate from σ_{tg} in trust games—even for reputation-type combinations that never occur on the equilibrium path (for example, a type who always free-rides but somehow becomes known as a contributor). For choosers, we must verify that σ_{ch} is optimal for every possible reputation ω, including those that never arise on the equilibrium path (for example, $(\text{unjustified free-rider}, \text{cheater})$ when choosers always distrust unjustified free-riders).

We define a reputation ω as *possible* if it can arise with positive probability when signalers play as prescribed by the strategy profile, assuming they reach intermediary reputations and are trusted where needed (even if equilibrium strategies would prevent reaching those reputations). Choosers' beliefs about types must be derived from Bayes' rule for all possible reputations, and may be specified arbitrarily otherwise.

This definition ensures that choosers form meaningful beliefs as widely as possible. For example, the reputation $(\text{contributor}, \text{unobserved})$ is only impossible if the probability of contribution is equal to 0, which requires that every type, save perhaps for a trivial subset of types $S \subseteq \Delta, \mathbb{P}(S) = 0$, initially free-rides even under normal circumstances. Similarly, the reputation $(\text{contributor}, \text{cheater})$ is only impossible if the corresponding actions are completely incompatible.

We focus on PBEs which allow for cooperation—contributions to the public good and dyadic cooperation in trust games—at any history, using the below definition.

Definition: Cooperative Equilibria

A PBE is cooperative if choosers' trust decisions incentivize signaler cooperative actions in any subgame; that is, if:

- Choosers trust contributors and distrust unjustified free-riders;
- Choosers trust reciprocators and distrust cheaters.

Following this definition, reciprocation is incentivized in every subgame. Regardless of public goods game reputation, a signaler who reciprocates incurs cost c in the current round but secures trust from the next chooser. Were choosers to behave differently, signalers would have no incentive to reciprocate. For example, were choosers to trust given both $(\text{contributor}, \text{reciprocator})$ and $(\text{contributor}, \text{cheater})$, signalers with reputation $(\text{contributor}, \cdot)$ would never reciprocate in a PBE, since doing so would incur cost c without improving their chances of being trusted.

This definition also enables contributions to the public good. Since contributing is always costly, choosers must also discriminate based on public goods game reputations for contribution to occur in equilibrium. They must at minimum trust given the most favorable piece of information—i.e., trust contributors—and distrust given the least favorable one—i.e., distrust unjustified free-riders. This leaves only one degree of freedom: whether to trust justified free-riders.

As detailed in Section 2, we obtain two cooperative equilibria—the flexible and the rigid norm—which differ in how they treat this ambiguous reputation. We show that the model's PBEs are either outcome-equivalent to one of these two equilibria, or do not involve any contributions to the public good.

2 Cooperative equilibria

We characterize the model's two cooperative equilibria: the flexible and the rigid norm. For each, we provide the full strategy profile and domain of existence in the general case and under the parameter values used in the main text. Other PBEs are either outcome-equivalent to one of these two equilibria, or do not involve any contributions to the public good. We also characterize these outcome-equivalent PBEs and compare their domains with those of the flexible and rigid norms.

2.1 Flexible norm

2.1.1 General results

Proposition 2.1: Flexible norm

Under the flexible norm, choosers trust justified free-riders. In addition, they trust contributors and reciprocators, while distrusting unjustified free-riders and cheaters. Whatever their reputation, signalers reciprocate choosers' trust if and only if their discount factor δ satisfies:

$$\delta \geq \delta_{\text{recip.}} \equiv \frac{c}{b}. \tag{2.1}$$

Signalers never contribute under hardship, whatever their discount factor δ. Under normal conditions, they contribute if and only if δ satisfies:

$$\delta \geq \delta_{\text{normal}}^{\text{flex.}} \equiv \min\{\frac{\gamma}{(1-\varepsilon)b}, \frac{\gamma}{\gamma+(1-\varepsilon)(b-c)}\}. \tag{2.2}$$

This equilibrium exists if and only if:

$$\mathbb{P}(\delta \geq \delta_{\text{recip.}} \mid \delta \geq \delta_{\text{normal}}^{\text{flex.}}) \geq \frac{c}{b}, \tag{2.3}$$

$$\mathbb{P}(\delta \geq \delta_{\text{recip.}} \mid \delta < \delta_{\text{normal}}^{\text{flex.}}) < \frac{c}{b}, \tag{2.4}$$

$$\frac{p\,\mathbb{P}(\delta \geq \delta_{\text{recip.}}) + (1-p)\varepsilon\,\mathbb{P}(\delta_{\text{recip.}} \leq \delta < \delta_{\text{normal}}^{\text{flex.}})}{p + (1-p)\varepsilon\,\mathbb{P}(\delta < \delta_{\text{normal}}^{\text{flex.}})} \geq \frac{c}{b}. \tag{2.5}$$

We prove this proposition in Appendix A. The proof proceeds in three steps. First, we establish that signalers reciprocate trust if and only if $\delta \geq c/b$ in any cooperative equilibrium, regardless of their current reputation (condition (2.1)). Second, we characterize contribution behavior in the public goods game. Under normal conditions, reciprocators contribute if $\delta \geq \frac{\gamma}{\gamma+(1-\varepsilon)(b-c)}$ and cheaters contribute if $\delta \geq \frac{\gamma}{(1-\varepsilon)b}$, yielding condition (2.2). Signalers never contribute under hardship since trust is already guaranteed. Third, we verify that choosers have no profitable deviations: contributors and justified free-riders must be sufficiently likely to reciprocate (conditions (2.3) and (2.5)), whereas unjustified free-riders must be sufficiently unlikely to do so (condition 2.4).

2.1.2 Main text parametrization

In the main text, we concentrate on the case where:

$$\frac{\gamma}{1-\varepsilon} < c < \gamma_H.$$

The left inequality ensures that contribution is easier than reciprocation, even after accounting for moral wiggle room enabled by the flexible norm. Under this condition, we have:

$$\delta_{\text{normal}}^{\text{flex.}} = \frac{\gamma}{(1-\varepsilon)b} < \frac{c}{b} = \delta_{\text{recip.}}.$$

Signalers can then be divided into three. Patient signalers ($\delta \geq \frac{c}{b}$) reciprocate and contribute under normal conditions; impatient signalers ($\delta < \frac{\gamma}{(1-\varepsilon)b}$) cheat and free-ride; while intermediate signalers

($\frac{\gamma}{(1-\varepsilon)b} \leq \delta < \frac{c}{b}$) cheat and contribute under normal conditions. (See Supplementary Figure 3 for signaler behavior under all parameter cases.)

In particular, there are no signalers that free-ride and reciprocate ($\mathbb{P}(\delta_{\text{recip.}} \leq \delta < \delta^{\text{flex.}}_{\text{normal}}) = 0$). This simplifies the expression for condition (2.5), which, for the sake of generality, incorporates the possibility of such types. In addition, condition (2.3) is implied by (2.5), since trusting justified free-riders is more difficult than trusting contributors; and condition (2.4) automatically holds, since all free-riders cheat.

We deduce:

Corollary 2.1: Flexible norm with main text parametrization

When

$$\frac{\gamma}{1-\varepsilon} < c < \gamma_H,$$

the threshold for contribution under normal conditions becomes:

$$\delta^{\text{flex.}}_{\text{normal}} = \frac{\gamma}{(1-\varepsilon)b}. \tag{2.2'}$$

The flexible norm then exists if and only if:

$$\frac{p\,\mathbb{P}(\delta \geq \delta_{\text{recip.}})}{p + (1-p)\varepsilon\,\mathbb{P}(\delta < \delta^{\text{flex.}}_{\text{normal}})} \geq \frac{c}{b}. \tag{2.5'}$$

2.2 Rigid norm

2.2.1 General result

Proposition 2.2: Rigid norm

Under the rigid norm, choosers distrust justified free-riders. In addition, they trust contributors and reciprocators, while distrusting unjustified free-riders and cheaters. Whatever their reputation, signalers reciprocate choosers' trust if and only if their discount factor δ satisfies:

$$\delta \geq \delta_{\text{recip.}} = \frac{c}{b}. \tag{2.1}$$

Under normal conditions, signalers contribute if and only if δ satisfies:

$$\delta \geq \delta^{\text{rigid}}_{\text{normal}} \equiv \min\{\frac{\gamma}{b}, \frac{\gamma}{\gamma + b - c}\}. \tag{2.6}$$

Under hardship, signalers contribute if and only if δ satisfies:

$$\delta \geq \delta^{\text{rigid}}_{\text{hardship}} \equiv \min\{\frac{\gamma_H}{b}, \frac{\gamma_H}{\gamma_H + b - c}\}. \tag{2.7}$$

This equilibrium exists if and only if:

$$\frac{p\,\mathbb{P}(\delta \geq \max\{\delta_{\text{recip.}}, \delta^{\text{rigid}}_{\text{hardship}}\}) + (1-p)\,\mathbb{P}(\delta \geq \max\{\delta_{\text{recip.}}, \delta^{\text{rigid}}_{\text{normal}}\})}{p\,\mathbb{P}(\delta \geq \delta^{\text{rigid}}_{\text{hardship}}) + (1-p)\,\mathbb{P}(\delta \geq \delta^{\text{rigid}}_{\text{normal}})} \geq \frac{c}{b}, \tag{2.8}$$

$$\mathbb{P}(\delta \geq \delta_{\text{recip.}} \mid \delta < \delta^{\text{rigid}}_{\text{normal}}) < \frac{c}{b}, \tag{2.9}$$

$$\frac{p\,\mathbb{P}(\delta_{\text{recip.}} \leq \delta < \delta^{\text{rigid}}_{\text{hardship}}) + (1-p)\varepsilon\,\mathbb{P}(\delta_{\text{recip.}} \leq \delta < \delta^{\text{rigid}}_{\text{normal}})}{p\,\mathbb{P}(\delta < \delta^{\text{rigid}}_{\text{hardship}}) + (1-p)\varepsilon\,\mathbb{P}(\delta < \delta^{\text{rigid}}_{\text{normal}})} < \frac{c}{b}. \tag{2.10}$$

We prove this proposition in Appendix A. As with the flexible norm, signalers reciprocate if and only if $\delta \geq c/b$ (condition (2.1)). Under normal conditions, reciprocators contribute if $\delta \geq \frac{\gamma}{\gamma+b-c}$ and cheaters if $\delta \geq \frac{\gamma}{b}$ (condition (2.6); note that these thresholds can be obtained by setting $\varepsilon = 0$ in the flexible norm thresholds). Unlike the flexible norm, trust is not guaranteed under hardship, so analogous thresholds apply there, with γ_H replacing γ (condition (2.7)). We obtain a PBE when choosers have no profitable

deviations: contributors must be sufficiently likely to reciprocate (condition (2.8)), whereas both types of free-riders must be sufficiently unlikely to do so (conditions (2.9) and (2.10)).

2.2.2 Main text parametrization

In the main text, we concentrate on the case where:

$$\frac{\gamma}{1-\varepsilon} < c < \gamma_H.$$

The left inequality implies

$$\gamma < c,$$

ensuring that contribution under normal conditions is easier than reciprocation in the rigid norm. The right inequality ensures that contribution under hardship is tougher. Under this condition, we have:

$$\delta^{\text{rigid}}_{\text{normal}} = \frac{\gamma}{b} < \frac{c}{b} < \frac{\gamma_H}{\gamma_H + b - c} = \delta^{\text{rigid}}_{\text{hardship}}$$

Signalers can then be divided into four. Very patient signalers ($\delta \geq \frac{\gamma_H}{\gamma_H+b-c}$) reciprocate and contribute even under hardship; relatively patient signalers ($\frac{c}{b} \leq \delta < \frac{\gamma_H}{\gamma_H+b-c}$) reciprocate and contribute under normal conditions; relatively impatient signalers ($\frac{\gamma}{b} \leq \delta < \frac{c}{b}$) cheat and contribute under normal conditions; very impatient signalers ($\delta < \frac{\gamma}{b}$) cheat and free-ride. (See Supplementary Figure 4 for signaler behavior under all parameter cases.)

This simplifies the expression for conditions (2.8) and (2.10). In addition, condition (2.9) automatically holds, since all signalers who free-ride under normal conditions cheat.

We deduce:

Corollary 2.2: Rigid norm with main text parametrization

When

$$\frac{\gamma}{1-\varepsilon} < c < \gamma_H,$$

the thresholds for contribution under normal conditions and hardship become:

$$\delta^{\text{rigid}}_{\text{normal}} = \frac{\gamma}{b}, \tag{2.6'}$$

$$\delta^{\text{rigid}}_{\text{hardship}} = \frac{\gamma_H}{\gamma_H + b - c}. \tag{2.7'}$$

The rigid norm then exists if and only if:

$$\frac{p\,\mathbb{P}(\delta \geq \delta^{\text{rigid}}_{\text{hardship}}) + (1-p)\,\mathbb{P}(\delta \geq \delta_{\text{recip.}})}{p\,\mathbb{P}(\delta \geq \delta^{\text{rigid}}_{\text{hardship}}) + (1-p)\,\mathbb{P}(\delta \geq \delta^{\text{rigid}}_{\text{normal}})} \geq \frac{c}{b}, \tag{2.8'}$$

$$\frac{p\,\mathbb{P}(\delta_{\text{recip.}} \leq \delta < \delta^{\text{rigid}}_{\text{hardship}})}{p\,\mathbb{P}(\delta < \delta^{\text{rigid}}_{\text{hardship}}) + (1-p)\varepsilon\,\mathbb{P}(\delta < \delta^{\text{rigid}}_{\text{normal}})} < \frac{c}{b}. \tag{2.10'}$$

2.3 Other equilibria

We analyze the model's other PBEs in Appendix B, which fall into two categories:

1. **Equilibria with initial contributions.** In these equilibria, signalers contribute to the public good with positive probability. All such equilibria are outcome-equivalent to either the flexible or the rigid norm: on the equilibrium path, behavior is identical, and differences arise only in off-path subgames following initial distrust. We detail these variants below, as they capture all cases in which there are contributions to the public good.

2. **Equilibria without initial contributions.** In these equilibria, signalers never contribute to the public good. We characterize these equilibria in Appendix B for completeness.

2.3.1 Flexible norm variant

Proposition 2.3: Flexible norm variant

There is one PBE that is outcome equivalent to the flexible norm. Players behave exactly as under the flexible norm, except after a signaler acquires reputation (unjustified free-rider, unobserved). In that off-path subgame, choosers always distrust and signalers always cheat.
This equilibrium exists if and only if:

$$\mathbb{P}(\delta \geq \delta_{\text{recip.}} \mid \delta \geq \delta^{\text{flex.}}_{\text{normal}}) \geq \frac{c}{b}, \tag{2.3}$$

$$\frac{p\,\mathbb{P}(\delta \geq \delta_{\text{recip.}}) + (1-p)\varepsilon\,\mathbb{P}(\delta_{\text{recip.}} \leq \delta < \delta^{\text{flex.}}_{\text{normal}})}{p + (1-p)\varepsilon\,\mathbb{P}(\delta < \delta^{\text{flex.}}_{\text{normal}})} \geq \frac{c}{b}. \tag{2.5}$$

We prove this proposition in Appendix B. In this variant of the flexible norm, distrust for unjustified free-riders is automatic, since cooperation trivially fails in the ensuing subgame. This removes condition (2.4), but does not change the domain of existence under our main text parameters, where such distrust was already automatic.

Corollary 2.3: Flexible norm variant with main text parametrization

When

$$\frac{\gamma}{1-\varepsilon} < c < \gamma_H,$$

the flexible norm variant exists under the same conditions as the flexible norm.

2.3.2 Rigid norm variants

Proposition 2.4: Rigid norm variants

There are three PBEs that are outcome-equivalent to the rigid norm. Players behave exactly as under the rigid norm, except after a signaler acquires reputation (justified free-rider, unobserved) or (unjustified free-rider, unobserved). In those off-path subgames, players either behave as in the rigid norm, or cooperation trivially fails: choosers always distrust and signalers always cheat.
One such equilibrium exists if and only if:

$$\frac{p\,\mathbb{P}(\delta \geq \max\{\delta_{\text{recip.}}, \delta^{\text{rigid}}_{\text{hardship}}\}) + (1-p)\,\mathbb{P}(\delta \geq \max\{\delta_{\text{recip.}}, \delta^{\text{rigid}}_{\text{normal}}\})}{p\,\mathbb{P}(\delta \geq \delta^{\text{rigid}}_{\text{hardship}}) + (1-p)\,\mathbb{P}(\delta \geq \delta^{\text{rigid}}_{\text{normal}})} \geq \frac{c}{b}. \tag{2.8}$$

We prove this proposition in Appendix B. In the most favorable variant of the rigid norm, distrust for unjustified and justified free-riders is automatic, since cooperation trivially fails in both ensuing subgames. This removes conditions (2.9) and (2.10), leading to an expanded domain of existence with our main text parametrization, which only made distrust for unjustified free-riders automatic.

Corollary 2.4: Rigid norm variants with main text parametrization

When

$$\frac{\gamma}{1-\varepsilon} < c < \gamma_H,$$

there exists a rigid norm variant if and only if:

$$\frac{p\,\mathbb{P}(\delta \geq \delta^{\text{rigid}}_{\text{hardship}}) + (1-p)\,\mathbb{P}(\delta \geq \delta_{\text{recip.}})}{p\,\mathbb{P}(\delta \geq \delta^{\text{rigid}}_{\text{hardship}}) + (1-p)\,\mathbb{P}(\delta \geq \delta^{\text{rigid}}_{\text{normal}})} \geq \frac{c}{b}, \tag{2.8'}$$

3 Beyond cooperative signals

Our model shows how rigid or flexible trust judgments can emerge when observers assess behavior in a collective action. Throughout Sections 1 and 2, we assume that contributions generate a public benefit $\beta > \gamma$, so that, under normal conditions, the initial stage is a standard public goods game in which cooperation is socially efficient. Here, we show that the equilibrium structure and the efficiency–robustness trade-off persist when $\beta = 0$—that is, when the initial stage reduces to a pure costly signaling problem, with no cooperative content.

3.1 β does not enter the equilibrium conditions

The parameter β does not appear in any of the conditions for existence of the flexible or rigid norms (conditions (2.3)–(2.5) and (2.8)–(2.10)). This is because signalers' contribution decisions are driven entirely by reputational incentives, not by the direct returns from the collective action. Under the flexible norm, sufficiently patient signalers incur the normal cost γ to secure trust—knowing that trust is only guaranteed with probability ε if they free-ride. Under the rigid norm, even more signalers do so under normal conditions (because free-riding guarantees distrust), and some also incur the high cost γ_H under hardship (which is no longer guaranteed for those who face hardship). In both cases, the incentive to contribute depends on patience, the cost of contribution under normal conditions or hardship, and the downstream value of trust—but not on β.

As a consequence, all results from Section 2 hold for any value of β, including $\beta = 0$. The two cooperative equilibria, their domains of existence (main text Figure 2), and the fraction of signalers who contribute under normal conditions (which increases under the rigid norm, shown in main text Figure 3a) are all unchanged. When $\beta = 0$, contributions are pure costly signals—such as loyalty tests, initiation rites, or other costly displays that reveal type without producing direct social benefits—but the logic of rigid and flexible assessment is the same.

3.2 Welfare comparison when $\beta = 0$

Although β does not affect the equilibrium structure, it enters the welfare comparison between the two norms. Under both norms, the expected per-capita payoff from the initial stage is:

$$\pi^{\text{flex}} = (1-p)\, F^{\text{flex}}_{\text{normal}}\, (\beta - \gamma), \tag{3.1}$$

$$\pi^{\text{rigid}} = (1-p)\, F^{\text{rigid}}_{\text{normal}}\, (\beta - \gamma) + p\, F^{\text{rigid}}_{\text{hardship}}\, (\beta - \gamma_H), \tag{3.2}$$

where $F^{\text{flex}}_{\text{normal}}$, $F^{\text{rigid}}_{\text{normal}}$, and $F^{\text{rigid}}_{\text{hardship}}$ denote the fractions of signalers who contribute in the relevant cases.

The rigid norm differs from the flexible norm in two ways. First, it lowers the contribution threshold under normal conditions ($\delta^{\text{rigid}}_{\text{normal}} < \delta^{\text{flex}}_{\text{normal}}$), inducing more signalers to contribute ($F^{\text{rigid}}_{\text{normal}} > F^{\text{flex}}_{\text{normal}}$). Second, it induces some signalers to contribute under hardship ($F^{\text{rigid}}_{\text{hardship}} > 0$), whereas the flexible norm does not. When $\beta > \gamma$, the first effect increases welfare (more socially valuable contributions) while the second decreases it (since $\beta < \gamma_H$, hardship contributions are socially wasteful). When $\beta = 0$, both effects decrease welfare—all contributions are pure cost—and the efficiency loss from rigidity is larger.

Supplementary Figure 2 illustrates this. Both panels show the per-capita payoff advantage of the flexible norm over the rigid norm, $\pi^{\text{flex}} - \pi^{\text{rigid}}$, across the domain where both norms are sustainable. The efficiency cost of rigidity is larger when $\beta = 0$ (panel b).

Note that, unlike main text Figure 3b, which normalizes the efficiency cost by the maximum achievable payoff $(1-p)\,(\beta - \gamma)$, here we report the raw payoff difference.

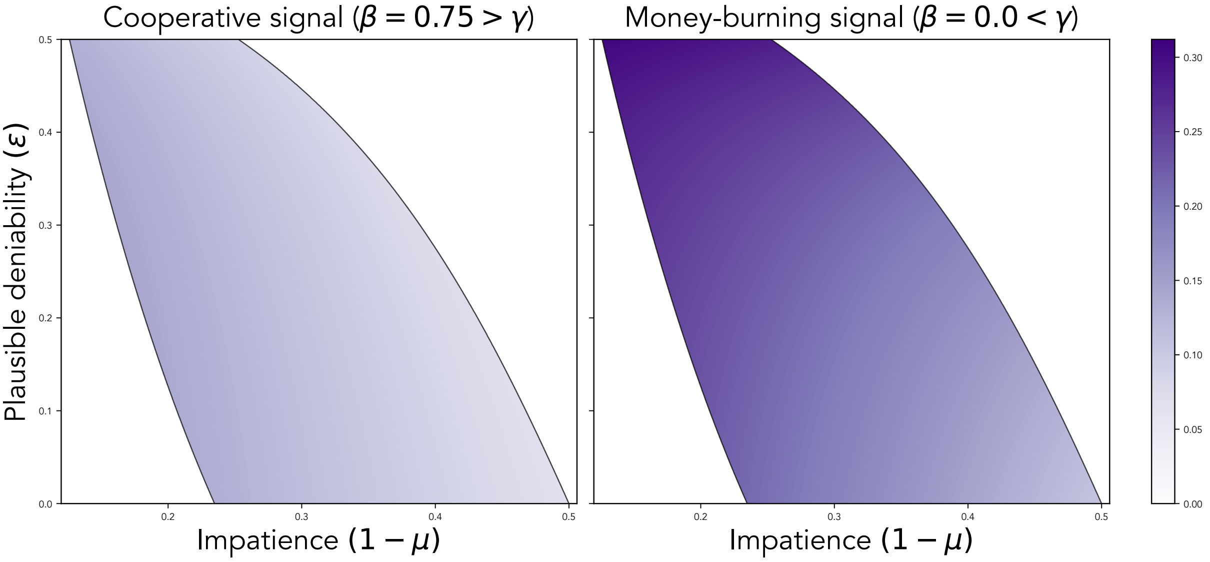

Supplementary Figure 2: **Efficiency cost of rigid norm: cooperative vs. money-burning signals.** Per-capita payoff advantage of the flexible norm over the rigid norm in the initial stage ($\pi^{\text{flex}} - \pi^{\text{rigid}}$). **(a)** Main-text parameters ($\beta = 0.75 > \gamma$): average payoff in the initial collective action is higher under the flexible norm (range: $0.07 - 0.14$, corresponding to $33\% - 71\%$ of the maximum achievable payoff). **(b)** $\beta = 0 < \gamma$: the efficiency cost of rigidity is larger when contributions are pure costly signals with no social benefits (range: $0.11 - 0.31$). The domain where both norms are sustainable—and all behavioral thresholds—are identical across both panels, since β does not enter any equilibrium condition. Parameters: $c = 1, b = 2, \gamma = 0.5, \beta = 0.75, \gamma_H = 2, p = 0.2$. We vary impatience $1 - \mu$ between 0 and 0.5 and plausible deniability ε between 0 and $1 - \frac{\gamma}{c} = 0.5$, ensuring that $\gamma/(1-\varepsilon) < c$ holds throughout.

References

[1] Drew Fudenberg and Jean Tirole. *Game Theory.* Cambridge, MA: MIT Press, 1991.

A Demonstrations for the cooperative equilibria

We characterize the model's cooperative equilibria, and derive their domains of existence.

A.1 Preliminary: continuation payoffs

In section 1, we define the lifetime payoff of a signaler of type δ, $U_{\text{sgl}}(\sigma \mid \delta)$. Here, we define signaler continuation payoffs, to aid with our proofs.

For any round $t \geq 1$, let $U^t_{\text{sgl}}(a_t \mid \delta, \omega)$ be a signaler's continuation payoff starting from round t, given that they have type $\delta \in \Delta$ and reputation $\omega = (\omega_{\text{pgg}}, \omega_{\text{tg}}) \in \Omega$, depending on the action $a_t = \sigma(\delta, \omega) \in \{\text{reciprocate}, \text{cheat}\}$ they would play if trusted this round. We omit σ from this notation for concision.

If the signaler has a reputation that leads to being distrusted—i.e., for a cooperative equilibrium, if they are known to be a cheater—they earn null payoff in this round, and also null payoff in later rounds, because signalers cannot change their reputation without being trusted (and choosers play the same pure strategy). In other words:

$$U^t_{\text{sgl}}(a_t \mid \delta, \omega) \equiv 0, \qquad \forall \omega \in \sigma^{-1}_{\text{ch}}(\text{distrust}).$$

If in contrast the signaler is to be trusted, their continuation payoff depends on their action a_t. By reciprocating, the signaler incurs cost c and becomes a reciprocator, acquiring reputation $\omega^{\text{rec.}} = (\omega_{\text{pgg}}, \text{reciprocator})$. Their continuation payoff is then:

$$U^t_{\text{sgl}}(\text{reciprocate} \mid \delta, \omega) \equiv (1-\delta) \times (b - c) + \delta \times U^{t+1}_{\text{sgl}}(\sigma_{\text{sgl}}(\delta, \omega^{\text{rec.}}) \mid \delta, \omega^{\text{rec.}}), \qquad \forall \omega \in \sigma^{-1}_{\text{ch}}(\text{trust}),$$

which notably depends on the action $a_{t+1} = \sigma_{\text{sgl}}(\delta, \omega^{\text{rec.}})$ that the signaler will play in the next round given that they reciprocate in the current one, and whether choosers trust given $\omega^{\text{rec.}}$. In contrast, by

cheating, the signaler incurs no cost and becomes a cheater, acquiring reputation $\omega^{\text{ch.}} = (\omega_{\text{pgg}}, \text{cheater})$. In this case, their continuation payoff is:

$$U^t_{\text{sgl}}(\text{cheat} \mid \delta, \omega) \equiv (1-\delta) \times b + \delta \times U^{t+1}_{\text{sgl}}(\sigma_{\text{sgl}}(\delta, \omega^{\text{ch.}}) \mid \delta, \omega^{\text{ch.}}), \qquad \forall \omega \in \sigma^{-1}_{\text{ch}}(\text{trust}).$$

A.2 Trust game behavior

We begin by showing that signalers reciprocate based only on their type in a cooperative equilibrium.

Lemma A.1: Trust game strategy σ_{tg}

In a cooperative equilibrium, whatever their current reputation, signalers reciprocate choosers' trust if and only if their discount factor δ satisfies:

$$\delta \geq \delta_{\text{recip.}} = \frac{c}{b}. \tag{2.1}$$

Proof. Consider a cooperative equilibrium. By definition, choosers discriminate based on reciprocation behavior in any subgame, trusting given any reputation of the form $(\cdot, \text{reciprocator})$ and distrusting given any reputation of the form $(\cdot, \text{cheater})$.

To derive signalers' trust game strategy, we must consider what happens after a signaler is trusted, obtaining an opportunity to act, given any type $\delta \in \Delta$ and reputation $\omega \in \Omega$—even in scenarios that are not supposed to occur.

If the signaler reciprocates, they incur cost c and acquire reputation $\omega^{\text{rec.}}$, obtaining continuation payoff $U^t_{\text{sgl}}(\text{reciprocate} \mid \delta, \omega)$, as defined above. If they cheat, they incur no cost and acquire reputation $\omega^{\text{ch.}}$, obtaining continuation payoff $U^t_{\text{sgl}}(\text{cheat} \mid \delta, \omega)$.

Since we are in a PBE, and given our tie-breaking rule, the signaler reciprocates if and only if:

$$U^t_{\text{sgl}}(\text{cheat} \mid \delta, \omega) \leq U^t_{\text{sgl}}(\text{reciprocate} \mid \delta, \omega),$$

that is, if and only if:

$$(1-\delta)\, b + \delta\, U^{t+1}_{\text{sgl}}(\sigma_{\text{sgl}}(\delta, \omega^{\text{ch.}}) \mid \delta, \omega^{\text{ch.}}) \leq (1-\delta)\,(b-c) + \delta\, U^{t+1}_{\text{sgl}}(\sigma_{\text{sgl}}(\delta, \omega^{\text{rec.}}) \mid \delta, \omega^{\text{rec.}}).$$

Re-arranging, the above condition is equivalent to:

$$(1-\delta)\, c \leq \delta\, (U^{t+1}_{\text{sgl}}(\sigma_{\text{sgl}}(\delta, \omega^{\text{rec.}}) \mid \delta, \omega^{\text{rec.}}) - U^{t+1}_{\text{sgl}}(\sigma_{\text{sgl}}(\delta, \omega^{\text{ch.}}) \mid \delta, \omega^{\text{ch.}}). \tag{A.1}$$

Importantly, this condition does not depend on the signaler's current reputation ω—whatever the current reputation, the signaler reciprocates the current chooser's trust in a PBE if and only if the future value (i.e. discounted by their δ) of achieving reputation $\omega^{\text{rec.}}$ rather than reputation $\omega^{\text{ch.}}$ is larger than the immediate cost of cooperation c.

Due to how reputations are updated (a signaler's public goods game reputation remains unchanged from round 1), this means that signalers adopt a stationary strategy in every subgame: from round 1, they either consistently reciprocate or always cheat when trusted, depending only on their type and potentially, the subgame—i.e., their public goods game reputation.

What's more, in a cooperative equilibrium, choosers treat reputation the same in every subgame. (This stands in contrast to non-cooperative equilibria, as we will see below.) As a result, signalers' trust game strategy depends only on their type: a (potentially empty) subset $S \subseteq \Delta$ of types satisfy condition (A.1) and always reciprocates, and its complement $\Delta \setminus S$ does not satisfy this condition and always cheats.

Finally, in a cooperative equilibrium, choosers distrust cheaters; for any type, we have:

$$U^{t+1}_{\text{sgl}}(\sigma_{\text{sgl}}(\delta, \omega^{\text{ch.}}) \mid \delta, \omega^{\text{ch.}}) = 0.$$

Consider a type $\delta \in S$. A signaler of this type who is trusted given reputation ω, reciprocates, obtaining reputation $\omega^{\text{rec.}}$, following which the signaler is trusted again in the next round, reciprocating again, and maintaining $\omega^{\text{rec.}}$. In this case, the signaler's continuation payoff from round $t+1$, after reaching a reciprocator reputation is:

$$U^{t+1}_{\text{sgl}}(\sigma_{\text{sgl}}(\delta, \omega^{\text{rec.}}) \mid \delta, \omega^{\text{rec.}}) = (1-\delta) \sum_{t' \geq 0} \delta^{t'} (b-c) = b-c.$$

Replacing, condition (A.1) becomes:

$$(1-\delta)c \leq \delta\,(b-c-0).$$

Subtracting δc on both sides and dividing by $b>0$, we equivalently obtain:

$$\frac{c}{b} \leq \delta.$$

This proves that S is non-trivial, and that signalers always reciprocate if and only if their type satisfies condition (2.1). □

A.3 Flexible norm

A.3.1 Trust game behavior

Under the flexible norm, choosers trust justified free-riders. Since the flexible norm is a cooperative PBE, by definition, they also trust contributors and reciprocators and distrust unjustified free-riders and cheaters.

A.3.2 Public goods game behavior

We turn to signalers' behavior in the initial public goods game. We distinguish reciprocating ($\delta \geq \frac{c}{b}$) and cheating ($\delta < \frac{c}{b}$) types.

Lemma A.2: Signaler public goods game strategy $\sigma_{\mathbf{pgg}}$

Under a flexible norm, signalers never contribute under hardship.
Under normal conditions, reciprocating types ($\delta \geq \frac{c}{b}$) contribute if and only if:

$$\delta \geq \delta^{\text{flex.}}_{\text{normal, rec.}} \equiv \frac{\gamma}{\gamma + (1-\varepsilon)(b-c)}. \tag{A.2}$$

In particular, every reciprocator contributes under normal conditions if $\varepsilon \leq 1 - \frac{\gamma}{c}$.
Cheating types ($\delta < \frac{c}{b}$) contribute if and only if:

$$\delta \geq \delta^{\text{flex.}}_{\text{normal, ch.}} \equiv \frac{\gamma}{(1-\varepsilon)b}. \tag{A.3}$$

In particular, every cheater free-rides under normal conditions if $\varepsilon \geq 1 - \frac{\gamma}{c}$.

Proof. Consider a cooperative equilibrium in which choosers trust justified free-riders. Following Lemma A.1, signalers always reciprocate if sufficiently patient ($\delta \geq \delta_{\text{recip.}}$) and always cheat otherwise ($\delta < \delta_{\text{recip.}}$).

Signalers who initially face hardship can either incur cost γ_H to be known as a contributor, or incur no cost to be known as a justified free-rider. In both cases, they are certain to be trusted in round 1, following which their reciprocation behavior is determined by their type: there is no reputational benefit to contributing in this case. As a result, since we are in a PBE and $\gamma_H > 0$, all signalers free-ride under hardship.

There are at most four cases for signalers who face normal conditions, depending on whether they contribute now and whether they reciprocate later. Consider first a patient reciprocating type ($\delta \geq \delta_{\text{recip.}}$). By contributing, this signaler incurs cost γ and ensures future trust, which is maintained from round 1 through reciprocal cooperation with choosers. The signaler's lifetime payoff is then:

$$U(\text{contribute} \mid \text{normal}, \delta \geq \frac{c}{b}) = (1-\delta)(-\gamma + \frac{n_c}{n-1}\beta) + \delta(b-c),$$

depending on the number of other signalers who contribute, n_c.

By free-riding, the signaler saves on cost γ but risks being distrusted; trust is only obtained if the signaler mistakenly obtains a justified free-rider reputation, with probability ε. The signaler's lifetime payoff is then:

$$U(\text{free-ride} \mid \text{normal}, \delta \geq \frac{c}{b}) = (1-\delta)(0 + \frac{n_c}{n-1}\beta) + \varepsilon\delta(b-c).$$

Comparing between the two, we deduce that reciprocating types contribute under normal conditions if and only if:

$$(1-\delta)\gamma \leq (1-\varepsilon)\delta(b-c),$$

which, re-arranging, is equivalent to:

$$\delta \geq \frac{\gamma}{\gamma+(1-\varepsilon)(b-c)}.$$

In particular, this is true for every reciprocating type if:

$$\frac{c}{b} \geq \frac{\gamma}{\gamma+(1-\varepsilon)(b-c)},$$

which is equivalent to:

$$c\gamma + c(1-\varepsilon)(b-c) \geq \gamma b.$$

Subtracting by γc and dividing by $b-c>0$, we equivalently obtain:

$$c(1-\varepsilon) \geq \gamma,$$

which is equivalent to:

$$\varepsilon \leq 1-\frac{\gamma}{c}.$$

When plausible deniability is sufficiently low and $\gamma < c$ (without which this condition can never be satisfied), every reciprocator type initially contributes under normal conditions.

Consider now an impatient cheater type ($\delta < \delta_{\text{recip.}}$) who initially faces normal conditions. By contributing, the signaler incurs cost γ and ensures future trust, which is betrayed in round 1, and never recouped. The signaler's lifetime payoff is then:

$$U(\text{contribute} \mid \text{normal}, \delta < \frac{c}{b}) = (1-\delta)(-\gamma + \frac{n_c}{n-1}\beta) + \delta(1-\delta)b.$$

By free-riding, the signaler instead obtains:

$$U(\text{free-ride} \mid \text{normal}, \delta < \frac{c}{b}) = (1-\delta) \times (0 + \frac{n_c}{n-1}\beta) + \varepsilon\delta(1-\delta)b.$$

Comparing between the two, we deduce that cheating types contribute under normal conditions if and only if:

$$(1-\delta)\gamma \leq (1-\varepsilon)\delta b(1-\delta),$$

which, re-arranging, is equivalent to:

$$\delta \geq \frac{\gamma}{(1-\varepsilon)b}.$$

In particular, this is true for every cheating type if:

$$\frac{c}{b} \leq \frac{\gamma}{(1-\varepsilon)b},$$

which is equivalent to:

$$c(1-\varepsilon) \leq \gamma,$$

and therefore to:

$$\varepsilon \geq 1-\frac{\gamma}{c}.$$

When plausible deniability is high or $\gamma \geq c$ (in which case the above condition is always satisfied), every cheater type initially free-rides under normal conditions. □

A.3.3 Signaler strategy profile ($\sigma_{\mathbf{pgg}}, \sigma_{\mathbf{tg}}$)

From Lemmas A.1 and A.2, we deduce signalers' entire strategy profile. Signalers reciprocate depending on how their type compares to $\delta_{\text{recip.}}$. They never contribute under hardship, and contribute under normal conditions depending on how their type compares to either $\delta^{\text{flex.}}_{\text{normal, rec.}}$ or $\delta^{\text{flex.}}_{\text{normal, ch.}}$.

As represented in Supplementary Figure 3, and immediately deduced from Lemma A.2, public goods game strategy can be represented with the threshold

$$\delta^{\text{flex.}}_{\text{normal}} = \min\{\delta^{\text{flex.}}_{\text{normal, ch.}}, \delta^{\text{flex.}}_{\text{normal, rec.}}\},$$

which is equal to $\delta^{\text{flex.}}_{\text{normal, ch.}}$ if and only if $\varepsilon \leq 1-\frac{\gamma}{c}$.

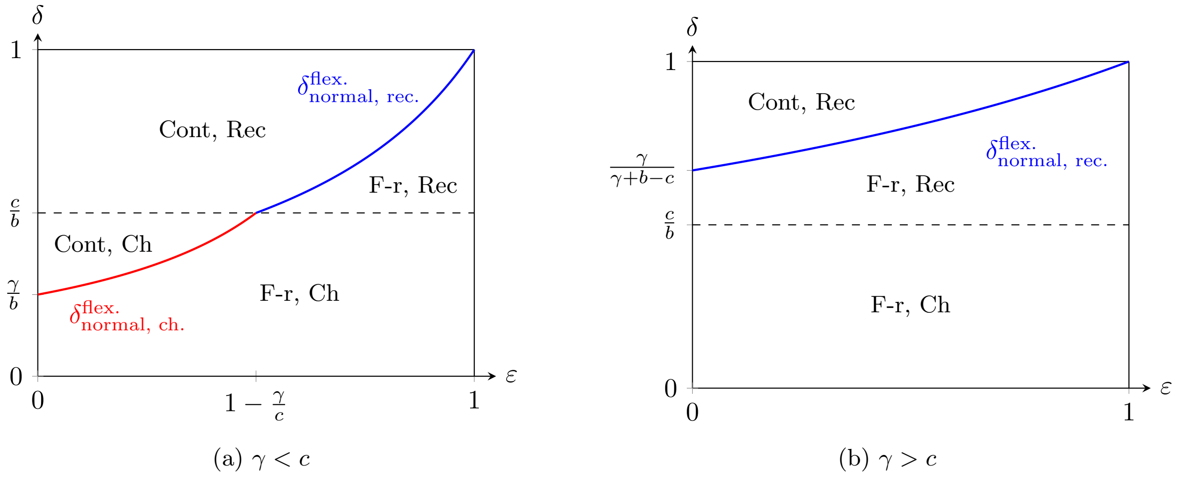

Supplementary Figure 3: Signaler behavior under the flexible norm, as a function of plausible deniability ε (x-axis) and type δ (y-axis). Signalers never contribute under hardship. Patient types contribute under normal conditions and reciprocate; impatient types free-ride and cheat. Intermediate type behavior depends on relative costs and plausible deniability. Labels: Cont = Contribute (under normal conditions), F-r = Free-ride, Rec = Reciprocate, Ch = Cheat. **(a)** When $\gamma < c$ (plotted: $\gamma = 0.5$, $c = 1$, $b = 2$): At low plausible deniability ($\varepsilon \leq 1 - \frac{\gamma}{c}$), intermediate types contribute and cheat; at high plausible deniability, they free-ride and reciprocate. **(b)** When $\gamma > c$ (plotted: $\gamma = 2$, $c = 1$, $b = 2$): Intermediate types always reciprocate and free-ride.

A.3.4 Flexible norm

Now that we have determined the entire strategy profile, we summarize our results, and derive the domain of existence of the flexible norm. This leads us to Proposition 2.1

Restatement of Proposition 2.1: Flexible norm

Under the flexible norm, choosers trust justified free-riders. In addition, they trust contributors and reciprocators, while distrusting unjustified free-riders and cheaters. Whatever their reputation, signalers reciprocate choosers' trust if and only if their discount factor δ satisfies:

$$\delta \geq \delta_{\text{recip.}} = \frac{c}{b}. \tag{2.1}$$

Signalers never contribute under hardship, whatever their discount factor δ. Under normal conditions, they contribute if and only if δ satisfies:

$$\delta \geq \delta^{\text{flex.}}_{\text{normal}} = \min\{\frac{\gamma}{(1-\varepsilon)b}, \frac{\gamma}{\gamma + (1-\varepsilon)(b-c)}\}. \tag{2.2}$$

This equilibrium exists if and only if:

$$\mathbb{P}(\delta \geq \delta_{\text{recip.}} \mid \delta \geq \delta^{\text{flex.}}_{\text{normal}}) \geq \frac{c}{b}, \tag{2.3}$$

$$\mathbb{P}(\delta \geq \delta_{\text{recip.}} \mid \delta < \delta^{\text{flex.}}_{\text{normal}}) < \frac{c}{b}, \tag{2.4}$$

$$\frac{p\,\mathbb{P}(\delta \geq \delta_{\text{recip.}}) + (1-p)\varepsilon\,\mathbb{P}(\delta_{\text{recip.}} \leq \delta < \delta^{\text{flex.}}_{\text{normal}})}{p + (1-p)\varepsilon\,\mathbb{P}(\delta < \delta^{\text{flex.}}_{\text{normal}})} \geq \frac{c}{b}. \tag{2.5}$$

Proof. Consider a cooperative equilibrium in which choosers trust justified free-riders, determining their strategy σ_{ch}. Following Lemmas A.1 and A.2, signalers' strategy profile $(\sigma_{\text{pgg}}, \sigma_{\text{tg}})$ is determined.

For the resulting strategy profile to be part of a PBE, beliefs must be derived using Bayes' rule whenever possible, and choosers' trust decisions for every reputation must be optimal given these beliefs.

We begin with initial reputations, of the form $(\omega_{\text{pgg}}, \text{unobserved})$, all three of which are attained. First, there are always contributors, who are signalers whose type satisfies $\delta \geq \delta^{\text{flex.}}_{\text{normal}}$ and who initially

face normal circumstances.

A chooser who trusts a contributor obtains on average:

$$u_{\text{ch}}(\text{trust} \mid \text{contributor}) = -c + \mathbb{P}(\text{reciprocate} \mid \text{contributor})\, b,$$

where the probability that the signaler will reciprocate, $\mathbb{P}(\text{reciprocate} \mid \text{contributor})$, satisfies:

$$\mathbb{P}(\text{reciprocate} \mid \text{contributor}) = \mathbb{P}(\delta \geq \delta_{\text{recip.}} \mid \text{contributor}),$$

since signalers reciprocate if and only if $\delta \geq \delta_{\text{recip.}}$. Since the reputation is possible, this conditional probability is derived using Bayes' rule:

$$\mathbb{P}(\text{reciprocate} \mid \text{contributor}) = \frac{\mathbb{P}(\delta \geq \delta_{\text{recip.}}, \text{contributor})}{\mathbb{P}(\text{contributor})},$$

yielding:

$$\mathbb{P}(\text{reciprocate} \mid \text{contributor}) = \frac{\mathbb{P}(\delta \geq \delta_{\text{recip.}},\, \delta \geq \delta^{\text{flex.}}_{\text{normal}},\, \text{normal conditions})}{\mathbb{P}(\delta \geq \delta^{\text{flex.}}_{\text{normal}},\, \text{normal conditions})}.$$

We obtain:

$$\mathbb{P}(\text{reciprocate} \mid \text{contributor}) = \frac{\mathbb{P}(\delta \geq \delta_{\text{recip.}},\, \delta \geq \delta^{\text{flex.}}_{\text{normal}})\,(1-p)}{\mathbb{P}(\delta \geq \delta^{\text{flex.}}_{\text{normal}})\,(1-p)}.$$

Dividing by $1-p$ and re-using Bayes' rule, we deduce:

$$\mathbb{P}(\text{reciprocate} \mid \text{contributor}) = \mathbb{P}(\delta \geq \delta_{\text{recip.}} \mid \delta \geq \delta^{\text{flex.}}_{\text{normal}}).$$

By deviating to distrusting in this scenario, the chooser would obtain 0 with certainty. This deviation isn't beneficial if and only if:

$$-c + \mathbb{P}(\delta \geq \delta_{\text{recip.}} \mid \delta \geq \delta^{\text{flex.}}_{\text{normal}})\, b \geq 0,$$

which, re-arranging, yields condition (2.3).

Likewise, there are always unjustified free-riders, who are signalers with $\delta < \delta^{\text{flex.}}_{\text{normal}}$ who initially faced normal circumstances and were unsuccessful in disguising these circumstances.

The probability that an unjustified free-rider will reciprocate can be similarly expressed using Bayes' rule:

$$\mathbb{P}(\text{reciprocate} \mid \text{unjustified free-rider}) = \frac{\mathbb{P}(\delta \geq \delta_{\text{recip.}},\, \delta < \delta^{\text{flex.}}_{\text{normal}})\,(1-p)\,(1-\varepsilon)}{\mathbb{P}(\delta < \delta^{\text{flex.}}_{\text{normal}})\,(1-p)\,(1-\varepsilon)}.$$

Re-arranging, and using Bayes' rule once again, we deduce

$$\mathbb{P}(\text{reciprocate} \mid \text{unjustified free-rider}) = \mathbb{P}(\delta \geq \delta_{\text{recip.}} \mid \delta < \delta^{\text{flex.}}_{\text{normal}}),$$

and that choosers do not deviate to trusting in this scenario if and only if condition (2.4) holds. (Recall that we assume that choosers trust if indifferent with distrusting.)

Finally for initial reputations, justified free-riders are signalers with any δ who faced hardship, and also signalers with $\delta < \delta^{\text{flex.}}_{\text{normal}}$ who faced normal conditions but successfully disguised them as hardship. In other words

$$\mathbb{P}(\text{justified free-rider}) = p + (1-p)\,\varepsilon\,\mathbb{P}(\delta < \delta^{\text{flex.}}_{\text{normal}}).$$

Reciprocators, with type $\delta \geq \delta_{\text{recip.}}$, attain this reputation if they initially face hardship, or if they face normal conditions and their type also satisfies $\delta < \delta^{\text{flex.}}_{\text{normal}}$ (which might be impossible; these conditions are incompatible whenever $\varepsilon < 1 - \frac{\gamma}{c}$, as visible in Supplementary Figure 3). We have:

$$\mathbb{P}(\delta \geq \delta_{\text{recip.}}, \text{justified free-rider}) = p\,\mathbb{P}(\delta \geq \delta_{\text{recip.}}) + (1-p)\,\varepsilon\,\mathbb{P}(\delta_{\text{recip.}} \leq \delta < \delta^{\text{flex.}}_{\text{normal}}).$$

Using the same steps as above, we deduce that choosers have no beneficial deviation from trusting justified free-riders if and only if condition (2.5) holds.

To conclude, we move on to reputations of the form $(\omega_{\text{pgg}}, \text{reciprocator})$ or $(\omega_{\text{pgg}}, \text{cheater})$. There are two cases: either the corresponding actions are compatible, in which case there exist types that can attain this reputation and would display the same trust game behavior, making any chooser deviation immediately costly; or the actions are incompatible, in which case the reputation is impossible, in

which case we assign beliefs corresponding to the trust game behavior, making any chooser deviation immediately costly.

For example, (contributor, reciprocator) is always possible, and attained by signalers whose δ exceeds both thresholds. Given this reputation, choosers form beliefs corresponding to such types, and deduce that the signaler will reciprocate again with certainty, obtaining:

$$u_{\text{ch}}(\text{trust} \mid (\text{contributor}, \text{reciprocator})) = -c + 1 \times b = b - c.$$

Since $b - c > 0$, they cannot benefit by deviating to distrust.

Likewise, (unjustified free-rider, cheater) is always possible, and it is always beneficial to distrust given this reputation.

There are, however, always some impossible reputations. As visible in Supplementary Figure 3, (unjustified free-rider, reciprocator) is impossible when $\varepsilon \leq 1 - \frac{\gamma}{c}$ (all reciprocators contribute under normal conditions), and (contributor, cheater) is impossible when $\varepsilon \geq 1 - \frac{\gamma}{c}$ (all contributors reciprocate). For these impossible reputations, we assign beliefs consistent with the trust game behavior—i.e., beliefs corresponding to $\delta \geq \delta_{\text{recip.}}$ for any reciprocator reputation, and $\delta < \delta_{\text{recip.}}$ for any cheater reputation. With these beliefs, trust is optimal for reciprocators and distrust is optimal for cheaters.

Therefore, these six reputations impose no additional constraints. Conditions (2.3)–(2.5) are necessary and sufficient for the flexible norm to constitute a PBE. □

A.4 Rigid norm

A.4.1 Trust game behavior

Under the rigid norm, choosers distrust justified free-riders. Since the rigid norm is a cooperative PBE, by definition, they also trust contributors and reciprocators and distrust unjustified free-riders and cheaters. Following Lemma A.1, signalers always reciprocate trust if $\delta \geq \delta_{\text{recip.}}$, and always cheat if $\delta < \delta_{\text{recip.}}$.

Thus, both σ_{ch} and σ_{tg} are determined.

A.4.2 Public goods game behavior

We now turn to signalers' behavior in the initial public goods game, σ_{pgg}, the remaining part of the strategy profile to determine. We distinguish reciprocating ($\delta \geq \frac{c}{b}$) and cheating ($\delta < \frac{c}{b}$) types.

Lemma A.3: Signaler public goods game strategy σ_{pgg}

Under a rigid norm, reciprocating types who face normal conditions contribute if and only if:

$$\delta \geq \delta^{\text{rigid}}_{\text{normal, rec.}} = \frac{\gamma}{\gamma + b - c}. \tag{A.4}$$

Under hardship, these types contribute if and only if:

$$\delta \geq \delta^{\text{rigid}}_{\text{hardship, rec.}} = \frac{\gamma_H}{\gamma_H + b - c}. \tag{A.5}$$

Cheating types who face normal conditions contribute if and only if:

$$\delta \geq \delta^{\text{rigid}}_{\text{normal, ch.}} = \frac{\gamma}{b}. \tag{A.6}$$

Under hardship, they contribute if and only if:

$$\delta \geq \delta^{\text{rigid}}_{\text{hardship, rec.}} = \frac{\gamma_H}{b}. \tag{A.7}$$

In particular, every reciprocator contributes under normal conditions if $\gamma \leq c$, and contributes under hardship if $\gamma_H \leq c$. Every cheater free-rides under normal conditions if $\gamma \geq c$, and under hardship if $\gamma_H \geq c$.

Proof. Consider a cooperative equilibrium in which choosers distrust justified free-riders. Following Lemma A.1, signalers always reciprocate if sufficiently patient ($\delta \geq \delta_{\text{recip.}}$) and always cheat otherwise ($\delta < \delta_{\text{recip.}}$).

Consider a signaler who initially faces normal conditions. There are at most four cases, depending on whether they contribute now and whether they reciprocate later. Consider first a patient reciprocating type ($\delta \geq \delta_{\text{recip.}}$). By contributing, this signaler incurs cost γ and ensures future trust, which is maintained from round 1 through reciprocal cooperation with choosers. The signaler's lifetime payoff is then:

$$U(\text{contribute} \mid \text{normal}, \delta \geq \frac{c}{b}) = (1-\delta)(-\gamma + \frac{n_c}{n-1}\beta) + \delta(b-c),$$

depending on the number of other signalers who contribute, n_c.

By free-riding, the signaler saves on cost γ but ensures distrust, since choosers distrust both unjustified and justified free-riders in this scenario. The signaler's lifetime payoff is then:

$$U(\text{free-ride} \mid \text{normal}, \delta \geq \frac{c}{b}) = (1-\delta)(0 + \frac{n_c}{n-1}\beta) + 0.$$

Comparing between the two, we deduce that reciprocating types contribute under normal conditions if and only if:

$$(1-\delta)\gamma \leq \delta(b-c),$$

which, re-arranging, is equivalent to:

$$\delta \geq \frac{\gamma}{\gamma + b - c}.$$

In particular, this is true for every reciprocating type if:

$$\frac{c}{b} \geq \frac{\gamma}{\gamma + b - c},$$

which is equivalent to:

$$c\gamma + c(b-c) \geq \gamma b.$$

Subtracting by γc and dividing by $b - c > 0$, we equivalently obtain:

$$c \geq \gamma.$$

When $\gamma \leq c$, every reciprocator type initially contributes under normal conditions.

Consider now an impatient cheater type ($\delta < \delta_{\text{recip.}}$) who initially faces normal conditions. By contributing, the signaler incurs cost γ and ensures future trust, which is betrayed in round 1, and never recouped. The signaler's lifetime payoff is then:

$$U(\text{contribute} \mid \text{normal}, \delta < \frac{c}{b}) = (1-\delta)(-\gamma + \frac{n_c}{n-1}\beta) + \delta(1-\delta)b.$$

By free-riding, the signaler instead obtains:

$$U(\text{free-ride} \mid \text{normal}, \delta < \frac{c}{b}) = (1-\delta) \times (0 + \frac{n_c}{n-1}\beta) + 0.$$

Comparing between the two, we deduce that cheating types contribute under normal conditions if and only if:

$$(1-\delta)\gamma \leq \delta b(1-\delta),$$

which, dividing by $1 - \delta$, is equivalent to:

$$\delta \geq \frac{\gamma}{b}.$$

In particular, every cheater free-rides under normal conditions if $\gamma \geq c$.

To conclude, we consider a signaler who faces hardship, which increases the cost of contribution to γ_H, without changing anything else. Replacing γ with γ_H in the previous steps, we deduce that reciprocators contribute if and only if:

$$\delta \geq \frac{\gamma_H}{\gamma_H + b - c},$$

while cheaters contribute under hardship if and only if:

$$\delta \geq \frac{\gamma_H}{b}.$$

In particular, every reciprocator contributes under hardship if $c \geq \gamma_H$, and every cheater free-rides if $\gamma_H \geq c$.

□

A.4.3 Signaler strategy profile $(\sigma_{\mathbf{pgg}}, \sigma_{\mathbf{tg}})$

From Lemmas A.1 and A.3, we deduce signalers' entire strategy profile. As represented in Supplementary Figure 4, and immediately deduced from Lemma A.3, public goods game strategy can be represented with the thresholds

$$\begin{aligned} \delta^{\text{rigid}}_{\text{normal}} &= \min\{\delta^{\text{rigid}}_{\text{normal, ch.}}, \delta^{\text{rigid}}_{\text{normal, rec.}}\}, \\ \delta^{\text{rigid}}_{\text{hardship}} &= \min\{\delta^{\text{rigid}}_{\text{hardship, ch.}}, \delta^{\text{rigid}}_{\text{hardship, rec.}}\}. \end{aligned}$$

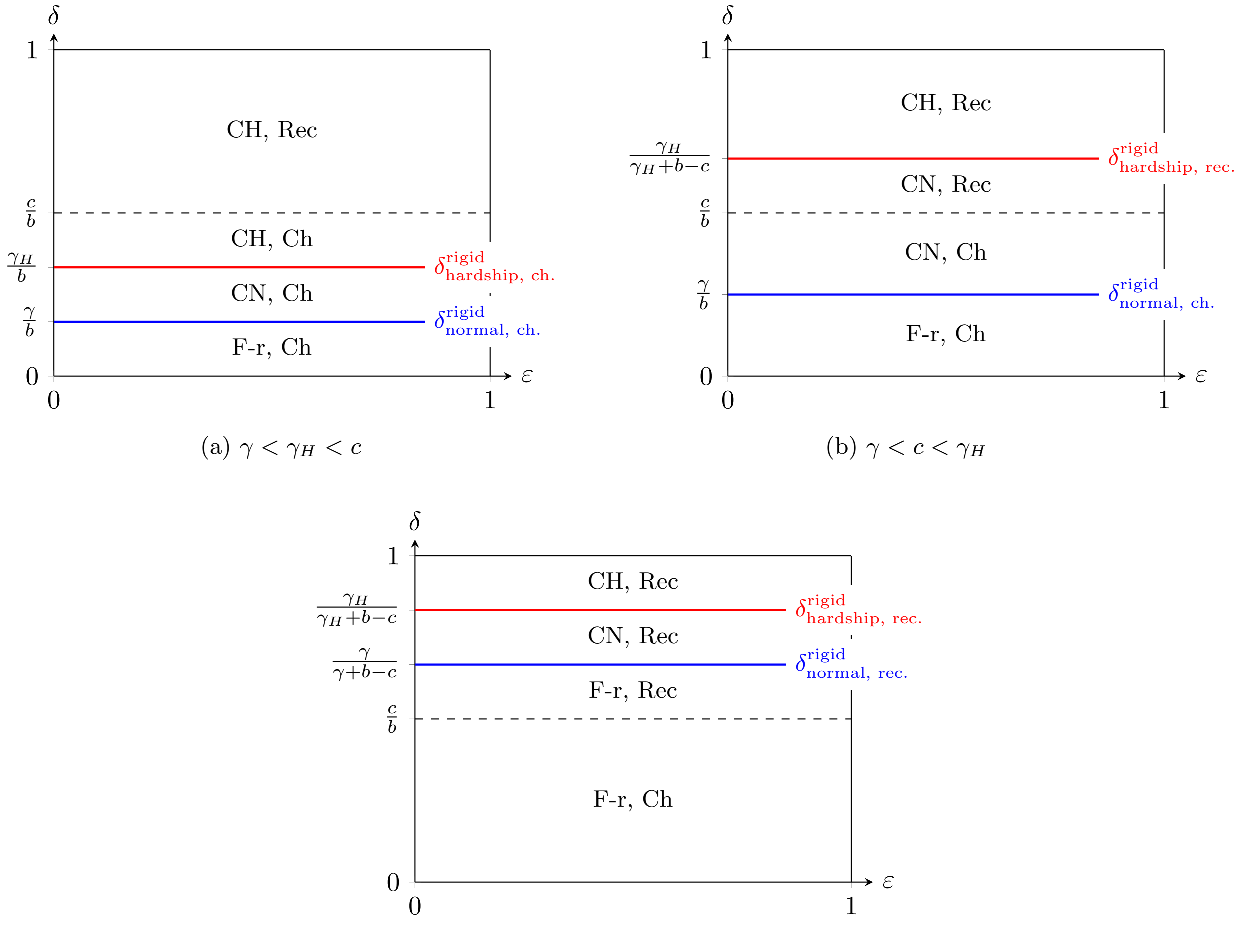

(a) $\gamma < \gamma_H < c$

(b) $\gamma < c < \gamma_H$

(c) $c < \gamma < \gamma_H$

Supplementary Figure 4: Signaler behavior under the rigid norm, as a function of type δ (y-axis). Unlike the flexible norm, thresholds are independent of plausible deniability ε (x-axis). Labels: CH = Contribute even under hardship, CN = Contribute only under normal conditions, F-r = Free-ride, Rec = Reciprocate, Ch = Cheat. **(a)** $\gamma < \gamma_H < c$ (plotted: $\gamma = 1/3$, $\gamma_H = 2/3$, $c = 1$, $b = 2$): Three types emerge among cheaters based on contribution behavior; all reciprocators contribute under hardship. **(b)** $\gamma < c < \gamma_H$ (plotted: $\gamma = 0.5$, $c = 1$, $\gamma_H = 2$, $b = 2$): Reciprocators either contribute under hardship or only under normal conditions; cheaters either contribute only under normal conditions or free-ride. **(c)** $c < \gamma < \gamma_H$ (plotted: $c = 1$, $\gamma = 2$, $\gamma_H = 5$, $b = 2$): All cheaters free-ride; three types of reciprocators based on contribution behavior.

A.4.4 Rigid norm

Now that we have determined the entire strategy profile, we summarize our results, and derive the domain of existence of the rigid norm. This leads us to Proposition 2.2

Restatement of Proposition 2.2: Rigid norm

Under the rigid norm, choosers distrust justified free-riders. In addition, they trust contributors and reciprocators, while distrusting unjustified free-riders and cheaters. Whatever their reputation, signalers reciprocate choosers' trust if and only if their discount factor δ satisfies:

$$\delta \geq \delta_{\text{recip.}} = \frac{c}{b}. \tag{2.1}$$

Under normal conditions, signalers contribute if and only if δ satisfies:

$$\delta \geq \delta^{\text{rigid}}_{\text{normal}} \equiv \min\{\frac{\gamma}{b}, \frac{\gamma}{\gamma + b - c}\}. \tag{2.6}$$

Under hardship, signalers contribute if and only if δ satisfies:

$$\delta \geq \delta^{\text{rigid}}_{\text{hardship}} \equiv \min\{\frac{\gamma_H}{b}, \frac{\gamma_H}{\gamma_H + b - c}\}. \tag{2.7}$$

This equilibrium exists if and only if:

$$\frac{p\,\mathbb{P}(\delta \geq \max\{\delta_{\text{recip.}}, \delta^{\text{rigid}}_{\text{hardship}}\}) + (1-p)\,\mathbb{P}(\delta \geq \max\{\delta_{\text{recip.}}, \delta^{\text{rigid}}_{\text{normal}}\})}{p\,\mathbb{P}(\delta \geq \delta^{\text{rigid}}_{\text{hardship}}) + (1-p)\,\mathbb{P}(\delta \geq \delta^{\text{rigid}}_{\text{normal}})} \geq \frac{c}{b}, \tag{2.8}$$

$$\mathbb{P}(\delta \geq \delta_{\text{recip.}} \mid \delta < \delta^{\text{rigid}}_{\text{normal}}) < \frac{c}{b}, \tag{2.9}$$

$$\frac{p\,\mathbb{P}(\delta_{\text{recip.}} \leq \delta < \delta^{\text{rigid}}_{\text{hardship}}) + (1-p)\varepsilon\,\mathbb{P}(\delta_{\text{recip.}} \leq \delta < \delta^{\text{rigid}}_{\text{normal}})}{p\,\mathbb{P}(\delta < \delta^{\text{rigid}}_{\text{hardship}}) + (1-p)\varepsilon\,\mathbb{P}(\delta < \delta^{\text{rigid}}_{\text{normal}})} < \frac{c}{b}. \tag{2.10}$$

Proof. Consider a cooperative equilibrium in which choosers distrust justified free-riders, determining their strategy σ_{ch}. Following Lemmas A.1 and A.3, signalers' strategy profile $(\sigma_{\text{pgg}}, \sigma_{\text{tg}})$ is determined.

For the resulting strategy profile to be part of a PBE, beliefs must be derived using Bayes' rule whenever possible, and choosers' trust decisions for every reputation must be optimal given these beliefs.

We begin with initial reputations, of the form $(\omega_{\text{pgg}}, \text{unobserved})$, all three of which are attained. First, there are always contributors, who are signalers with $\delta \geq \delta^{\text{rigid}}_{\text{normal}}$ who initially face normal circumstances, or signalers with $\delta \geq \delta^{\text{hardship}}_{\text{normal}}$ who face hardship.

A chooser who trusts a contributor obtains on average:

$$u_{\text{ch}}(\text{trust} \mid \text{contributor}) = -c + \mathbb{P}(\text{reciprocate} \mid \text{contributor})\, b,$$

where the probability that the signaler will reciprocate, $\mathbb{P}(\text{reciprocate} \mid \text{contributor})$, satisfies:

$$\mathbb{P}(\text{reciprocate} \mid \text{contributor}) = \mathbb{P}(\delta \geq \delta_{\text{recip.}} \mid \text{contributor}),$$

since signalers reciprocate if and only if $\delta \geq \delta_{\text{recip.}}$. Since the reputation is possible, this conditional probability is derived using Bayes' rule:

$$\mathbb{P}(\text{reciprocate} \mid \text{contributor}) = \frac{\mathbb{P}(\delta \geq \delta_{\text{recip.}}, \text{contributor})}{\mathbb{P}(\text{contributor})},$$

the expression on the right being equal to:

$$\frac{\mathbb{P}(\delta \geq \delta_{\text{recip.}}, ((\delta \geq \delta^{\text{rigid}}_{\text{normal}} \text{ and normal conditions}) \text{ or } (\delta \geq \delta^{\text{rigid}}_{\text{hardship}} \text{ and hardship})))}{\mathbb{P}((\delta \geq \delta^{\text{rigid}}_{\text{normal}} \text{ and normal conditions}) \text{ or } (\delta \geq \delta^{\text{rigid}}_{\text{hardship}} \text{ and hardship}))}.$$

We obtain:

$$\mathbb{P}(\text{reciprocate} \mid \text{contributor}) = \frac{(1-p)\,\mathbb{P}(\delta \geq \max\{\delta_{\text{recip.}}, \delta^{\text{rigid}}_{\text{normal}}\}) + p\,\mathbb{P}(\delta \geq \max\{\delta_{\text{recip.}}, \delta^{\text{rigid}}_{\text{hardship}}\})}{(1-p)\,\mathbb{P}(\delta \geq \delta^{\text{rigid}}_{\text{normal}}) + p\,\mathbb{P}(\delta \geq \delta^{\text{rigid}}_{\text{hardship}})}.$$

By deviating to distrusting in this scenario, the chooser would obtain 0 with certainty. This deviation isn't beneficial if and only if:

$$-c + \mathbb{P}(\delta \geq \delta_{\text{recip.}} \mid \text{contributor})\, b \geq 0,$$

which, re-arranging, yields condition (2.8).

More simply, there are always unjustified free-riders, who, as under the flexible norm, are signalers with $\delta < \delta_{\text{normal}}^{\text{rigid}}$ who initially faced normal circumstances and were unsuccessful in disguising these circumstances.

The probability that an unjustified free-rider will reciprocate can be expressed using Bayes' rule:

$$\mathbb{P}(\text{reciprocate} \mid \text{unjustified free-rider}) = \frac{\mathbb{P}(\delta \geq \delta_{\text{recip.}},\, \delta < \delta_{\text{normal}}^{\text{rigid}})\,(1-p)\,(1-\varepsilon)}{\mathbb{P}(\delta < \delta_{\text{normal}}^{\text{rigid}})\,(1-p)\,(1-\varepsilon)}.$$

Re-arranging, and using Bayes' rule once again, we deduce

$$\mathbb{P}(\text{reciprocate} \mid \text{unjustified free-rider}) = \mathbb{P}(\delta \geq \delta_{\text{recip.}} \mid \delta < \delta_{\text{normal}}^{\text{rigid}}),$$

and that choosers do not deviate to trusting in this scenario if and only if condition (2.9) holds.

Finally for initial reputations, justified free-riders are signalers with $\delta < \delta_{\text{hardship}}^{\text{rigid}}$ who faced hardship, or signalers with $\delta < \delta_{\text{normal}}^{\text{rigid}}$ who faced normal conditions but successfully disguised them as hardship. In other words

$$\mathbb{P}(\text{justified free-rider}) = p\,\mathbb{P}(\delta < \delta_{\text{hardship}}^{\text{rigid}}) + (1-p)\,\varepsilon\,\mathbb{P}(\delta < \delta_{\text{normal}}^{\text{rigid}}).$$

Reciprocators, with type $\delta \geq \delta_{\text{recip.}}$, attain this reputation under similar conditions:

$$\mathbb{P}(\delta \geq \delta_{\text{recip.}},\, \text{justified free-rider}) = p\,\mathbb{P}(\delta_{\text{recip.}} \leq \delta < \delta_{\text{hardship}}^{\text{rigid}}) + (1-p)\,\varepsilon\,\mathbb{P}(\delta_{\text{recip.}} \leq \delta < \delta_{\text{normal}}^{\text{rigid}}).$$

Using the same steps as above, we deduce that choosers have no beneficial deviation from distrusting justified free-riders if and only if condition (2.10) holds.

To conclude, we move on to reputations of the form $(\omega_{\text{pgg}}, \text{reciprocator})$ or $(\omega_{\text{pgg}}, \text{cheater})$. As with the flexible norm, there are two cases: either the corresponding actions are compatible, in which case there exist types that can attain this reputation and would display the same trust game behavior, making any chooser deviation immediately costly; or the actions are incompatible, in which case the reputation is impossible, in which case we assign beliefs corresponding to the trust game behavior, making any chooser deviation immediately costly.

For example, $(\text{contributor}, \text{reciprocator})$ is always possible, and is attained by signalers with $\delta \geq \delta_{\text{recip.}}$ and who either face hardship and satisfy the conditions for contribution under hardship, or face normal conditions and satisfy the conditions for contribution there. Given this reputation, choosers form beliefs corresponding to such types, and deduce that the signaler will reciprocate again with certainty, obtaining:

$$u_{\text{ch}}(\text{trust} \mid (\text{contributor}, \text{reciprocator})) = -c + 1 \times b = b - c.$$

Since $b - c > 0$, they cannot benefit by deviating to distrust.

Likewise, $(\text{unjustified free-rider}, \text{cheater})$ is always possible, and it is always beneficial to distrust given this reputation.

There are, however, always some impossible reputations. As visible in Supplementary Figure 4, $(\text{unjustified free-rider}, \text{reciprocator})$ is impossible when $\gamma < c$ (all reciprocators contribute under normal conditions), and $(\text{contributor}, \text{cheater})$ is impossible when $\gamma > c$ (all contributors reciprocate). For these impossible reputations, we assign beliefs consistent with the trust game behavior—i.e., beliefs corresponding to $\delta \geq \delta_{\text{recip.}}$ for any reciprocator reputation, and $\delta < \delta_{\text{recip.}}$ for any cheater reputation. With these beliefs, trust is optimal for reciprocators and distrust is optimal for cheaters.

Therefore, these six reputations impose no additional constraints. Conditions (2.8)–(2.10) are necessary and sufficient for the rigid norm to constitute a PBE. □

B Demonstrations for other equilibria

We characterize the model's other PBEs, distinguishing between those with and without initial contributions.

B.1 General results

We begin with three Lemmas that characterize chooser equilibrium behavior.

Lemma B.1: Discrimination following trust

In a PBE, if choosers trust given some initial reputation $(\omega_{\text{pgg}}, \text{unobserved})$, they must subsequently discriminate between reciprocators and cheaters: they trust given $(\omega_{\text{pgg}}, \text{reciprocator})$ and distrust given $(\omega_{\text{pgg}}, \text{cheater})$.

Proof. Consider a PBE where choosers trust given some initial reputation $(\omega_{\text{pgg}}, \text{unobserved})$. Since trust is costly, it must be incentivized by the signaler: there exists a positive-measure set of types that reciprocate trust given $(\omega_{\text{pgg}}, \text{unobserved})$.

Since reciprocation is costly for every type, it must be incentivized by subsequent trust. The only way to do so is for choosers to trust given $(\omega_{\text{pgg}}, \text{reciprocator})$ and distrust given $(\omega_{\text{pgg}}, \text{cheater})$. Any other pair of actions either provides no incentive to reciprocate (if trust is guaranteed regardless of behavior, or distrust is guaranteed regardless of behavior), or actively disincentivizes reciprocation (if cheaters are trusted). □

Lemma B.2: Two possibilities following distrust

In a PBE, if choosers distrust given some initial reputation $(\omega_{\text{pgg}}, \text{unobserved})$, one of two possible patterns emerges in the resulting subgame:

1. **Discriminating subgame**: Choosers trust given $(\omega_{\text{pgg}}, \text{reciprocator})$ and distrust given $(\omega_{\text{pgg}}, \text{cheater})$, and signalers reciprocate if and only if $\delta \geq \delta_{\text{recip.}}$.
2. **Non-discriminating subgame**: Choosers distrust given both $(\omega_{\text{pgg}}, \text{reciprocator})$ and $(\omega_{\text{pgg}}, \text{cheater})$, and all signaler types always cheat.

Proof. Consider a PBE where choosers distrust given some initial reputation $(\omega_{\text{pgg}}, \text{unobserved})$. Subsequent behavior is then off the equilibrium path.

There are two possibilities: either reciprocation is incentivized or it is not. If choosers trust given $(\omega_{\text{pgg}}, \text{reciprocator})$ and distrust given $(\omega_{\text{pgg}}, \text{cheater})$, then signalers in that subgame reciprocate if and only if $\delta \geq \delta_{\text{recip.}}$ (as established in Lemma A.1). Choosers' decisions are then optimal, since these reputations perfectly predict signaler behavior.

Otherwise, given any other pair of chooser actions, reciprocation is not incentivized, leading every signaler type to always cheat. As a result, the only optimal choice for choosers is to always distrust. □

Lemma B.3: Discrimination by contribution

In a PBE, if signalers contribute with positive probability, choosers must trust contributors and distrust unjustified free-riders.

Proof. Consider a PBE where signalers contribute with positive probability. There exists a positive-measure set of types that contribute under normal conditions (where costs are lower and reputational benefits the same as under hardship).

Since contribution is costly for every type, it must be incentivized by subsequent trust. Contributors must be trusted, and choosers must distrust at least one type of free-rider. We show that they must distrust unjustified free-riders.

Suppose that choosers distrust justified free-riders. Unjustified free-riders are signalers who faced normal conditions and free-rode, and can therefore be deduced to be too impatient to contribute under normal conditions. Justified free-riders, in contrast, faced either normal conditions or hardship, and are thus either of a similarly impatient type or a more patient one, since contribution under hardship is more expensive. In other words, the justified free-rider reputation is a better predictor of reciprocation than the unjustified free-rider reputation; in a PBE, if choosers distrust given the better predictor of reciprocation, they must also distrust given the worse predictor. Therefore, choosers must distrust unjustified free-riders. □

B.2 Equilibria with initial contributions

Lemma B.4: Other equilibria with contributions

Any PBE in which signalers contribute to the public good with positive probability is outcome-equivalent to either the flexible norm or the rigid norm. Behavior along the equilibrium path is identical to one of these two norms, with differences arising only in off-path subgames.

Restatement of Proposition 2.3: Flexible norm variant

There is one PBE that is outcome equivalent to the flexible norm. Players behave exactly as under the flexible norm, except after a signaler acquires reputation (unjustified free-rider, unobserved). In that off-path subgame, choosers always distrust and signalers always cheat.
This equilibrium exists if and only if:

$$\mathbb{P}(\delta \geq \delta_{\text{recip.}} \mid \delta \geq \delta^{\text{flex.}}_{\text{normal}}) \geq \frac{c}{b}, \tag{2.3}$$

$$\frac{p\,\mathbb{P}(\delta \geq \delta_{\text{recip.}}) + (1-p)\varepsilon\,\mathbb{P}(\delta_{\text{recip.}} \leq \delta < \delta^{\text{flex.}}_{\text{normal}})}{p + (1-p)\varepsilon\,\mathbb{P}(\delta < \delta^{\text{flex.}}_{\text{normal}})} \geq \frac{c}{b}. \tag{2.5}$$

Restatement of Proposition 2.4: Rigid norm variants

There are three PBEs that are outcome-equivalent to the rigid norm. Players behave exactly as under the rigid norm, except after a signaler acquires reputation (justified free-rider, unobserved) or (unjustified free-rider, unobserved). In those off-path subgames, players either behave as in the rigid norm, or cooperation trivially fails: choosers always distrust and signalers always cheat.
One such equilibrium exists if and only if:

$$\frac{p\,\mathbb{P}(\delta \geq \max\{\delta_{\text{recip.}}, \delta^{\text{rigid}}_{\text{hardship}}\}) + (1-p)\,\mathbb{P}(\delta \geq \max\{\delta_{\text{recip.}}, \delta^{\text{rigid}}_{\text{normal}}\})}{p\,\mathbb{P}(\delta \geq \delta^{\text{rigid}}_{\text{hardship}}) + (1-p)\,\mathbb{P}(\delta \geq \delta^{\text{rigid}}_{\text{normal}})} \geq \frac{c}{b}. \tag{2.8}$$

Proof. We prove the lemma and both propositions together. Consider a PBE where signalers contribute to the public good with positive probability. Following Lemma B.3, choosers trust contributors and distrust unjustified free-riders: based on whether they trust justified free-riders, their initial decisions are identical to those in the flexible or rigid norm.

In addition, in every subgame after initial trust, choosers discriminate and signalers reciprocate if and only if $\delta \geq \delta_{\text{recip.}}$ following Lemma B.1. Behavior along the equilibrium path is then identical to that of the flexible or rigid norm—any PBE with initial contributions is outcome-equivalent to either the flexible or the rigid norm.

The only remaining degrees of freedom concern unattained subgames following initial distrust. By Lemma B.2, there are two possibilities in each case. This leads to two outcome-equivalent equilibria in which choosers trust justified free-riders—the flexible norm and one variant in which the subgame after the unjustified free-rider reputation is non-discriminating. Likewise, there are four outcome-equivalent equilibria in which choosers distrust justified free-riders—the rigid norm and three variants—depending on whether the subgames after either free-rider label are discriminating or non-discriminating. ☐

B.3 Equilibria without initial contributions

Lemma B.5: Equilibria without contributions

There are two cases for PBEs without initial contributions.

Case 1. Choosers initially trust all signalers. Subsequently, in every subgame, they discriminate between reciprocators and cheaters, and signalers reciprocate trust if and only if $\delta \geq \delta_{\text{recip.}}$. A PBE obtains if and only if

$$\mathbb{P}(\delta \geq \delta_{\text{recip.}}) \geq \frac{c}{b}. \tag{B.1}$$

Case 2. Choosers initially distrust all signalers. Subsequently, in every subgame, there are two possibilities: either choosers always distrust and signalers always cheat, or choosers discriminate between reciprocators and cheaters, and signalers reciprocate trust if and only if $\delta \geq \delta_{\text{recip.}}$. All such cases constitute PBEs if and only if

$$\mathbb{P}(\delta \geq \delta_{\text{recip.}}) < \frac{c}{b}. \tag{B.1'}$$

Proof. Consider a PBE without initial contributions. Since every signaler type free-rides, all reputation labels become uninformative about type. Choosers must therefore either trust all signalers or distrust all signalers, depending on whether it is profitable on average to trust blindly; that is, on whether condition (B.1) or (B.1') holds.

Because contribution behavior does not affect subsequent trust, no signaler type pays the cost of contribution under either normal conditions or hardship.

Subsequently, Lemmas B.1 and B.2 apply. This leads to one PBE with initial trust and $2^3 = 8$ with initial distrust. In all cases, neither signalers nor choosers have profitable deviations. □
